# Differential responses of innate immunity triggered by different subtypes of influenza a viruses in human and avian hosts

**DOI:** 10.1186/s12920-017-0304-z

**Published:** 2017-12-21

**Authors:** Yingying Cao, Yaowei Huang, Ke Xu, Yuanhua Liu, Xuan Li, Ye Xu, Wu Zhong, Pei Hao

**Affiliations:** 1 Key Laboratory of Molecular Virology and Immunology, Institute Pasteur of Shanghai, University of Chinese Academy of Sciences, Shanghai, China; 2 Key Laboratory of Synthetic Biology, CAS Center for Excellence in Molecular Plant Sciences, Institute of Plant Physiology and Ecology, University of Chinese Academy of Sciences, Shanghai, China; 3 Department of Colorectal Surgery, Fudan University Shanghai Cancer Center, Shanghai, China; 4 National Engineering Research Center For the Emergence Drugs, Beijing Institute of Pharmacology and Toxicology, Beijing, 100850 China

**Keywords:** Influenza a virus, Innate immune response, Cytokines, Chemokines

## Abstract

**Background:**

Innate immunity provides first line of defense against viral infections. The interactions between hosts and influenza A virus and the response of host innate immunity to viral infection are critical determinants for the pathogenicity or virulence of influenza A viruses. This study was designed to investigate global changes of gene expression and detailed responses of innate immune systems in human and avian hosts during the course of infection with various subtypes of influenza A viruses, using collected and self-generated transcriptome sequencing data from human bronchial epithelial (HBE), human tracheobronchial epithelial (HTBE), and A549 cells infected with influenza A virus subtypes, namely H1N1, H3N2, H5N1 HALo mutant, and H7N9, and from ileum and lung of chicken and quail infected with H5N1, or H5N2.

**Results:**

We examined the induction of various cytokines and chemokines in human hosts infected with different subtypes of influenza A viruses. Type I and III interferons were found to be differentially induced with each subtype. H3N2 caused abrupt and the strongest response of IFN-β and IFN-λ, followed by H1N1 (though much weaker), whereas H5N1 HALo mutant and H7N9 induced very minor change in expression of type I and III interferons. Similarly, differential responses of other innate immunity-related genes were observed, including TMEM173, MX1, OASL, IFI6, IFITs, IFITMs, and various chemokine genes like CCL5, CX3CL1, and chemokine (C-X-C motif) ligands, SOCS (suppressors of cytokine signaling) genes. Third, the replication kinetics of H1N1, H3N2, H5N1 HALo mutant and H7N9 subtypes were analyzed, H5N1 HALo mutant was found to have the highest viral replication rate, followed by H3N2, and H1N1, while H7N9 had a rate similar to that of H1N1 or H3N2 though in different host cell type.

**Conclusion:**

Our study illustrated the differential responses of innate immunity to infections of different subtypes of influenza A viruses. We found the influenza viruses which induced stronger innate immune responses replicate slower than those induces weaker innate immune responses. Our study provides important insight into links between the differential innate immune responses from hosts and the pathogenicity/ virulence of different subtypes of influenza A viruses.

**Electronic supplementary material:**

The online version of this article (10.1186/s12920-017-0304-z) contains supplementary material, which is available to authorized users.

## Background

Influenza A viruses are major pathogens with potential to unleash epidemics and pandemics of respiratory disease in human and avian hosts. There have been repeated outbreaks of highly pathogenic avian influenza (HPAI) H5N1 in poultries and human infections associated with high mortality [1, 2]. H1N1 and H3N2 subtypes of influenza A viruses caused the pandemics in 1918 and 1968, respectively [3–5]. Recently, the H1N1 virus of swine origin (pH1N1) was the cause of 2009 pandemic [6]. Although influenza A viruses are mostly associated with mild and self-limiting symptom, the 2009 pH1N1, H5N1 and H7N9 incurred severe and fatal outcomes to infected individuals [6–8]. Some studies show that regulation of host immunity is largely dependent on the subtypes of the viruses. Avian-orgin viruses like H5N1, H7N9 were reported to suppress the innate immunity of cells while the human-seansonal influenza viruses stimulate the innate immunity [9–11]. However, the detailed mechanisms and the dynamic regulation of virus-host interaction among subtypes still need further investigation.

Influenza A viruses can initiate a strong innate immune response that is critical for defense against and clearing of the infections. The primary targets of influenza A infection are lung and bronchial epithelial cells that play a critical role in instigating the innate immune responses [12]. Innate immune responses to virus infection include sensing of viral proteins and nucleic acids, production of specific cytokines and chemokines, activation of complement cascade, etc. First, the innate immune system has the ability to recognize viral proteins or nucleic acids as invader, using a family of Pattern Recognition Receptors (PRRs). For example, viral double-stranded RNA (dsRNA) or single-stranded RNA (ssRNA) with a 5′-triphosphate that are typical products of viral replication, can be detected by the cytoplasmic retinoic acid-inducible gene I (RIG-I) like receptors (RLRs) [13–16] and Toll-like receptors (TLRs) [17]. The detection of viral RNAs leads to expression of antiviral genes, including interferons and pro-inflammatory chemokines that elicit an intracellular immune response to control virus infection [18, 19]. Signaling of viral infection through RLRs launches an immune response that is characterized by the transcriptional up-regulation of many antiviral molecules, including pro-inflammatory cytokines, chemokines like C-X-C motif ligands, C-C motif ligands, and IFN-α/β/λ, and IFN-stimulated genes like OASL (2′-5′-oligoadenylate synthetase-like) and MX-1 (MX Dynamin Like GTPase 1) [20].

Infection with some subtypes of influenza A viruses such as H5N1 and H7N9 can cause severe respiratory disease [21, 22]. Some studies have found that H5N1 and H7N9 inducted weaker innate immune response [9–11], but to our knowledge, none of these studies have quantified the change of different innate immune responses due to the limitation of technology. Recently, next-generation sequencing technology and transcriptome sequencing (RNA-seq) methods [23–25] enabled us to implement large scale study to analyze the induced expression of innate immunity-related genes in hosts infected with various subtypes of influenza A virus. In the current study, to examine the difference in innate immune responses to different subtypes of influenza A viruses, we collected from public data source, like National Center for Biotechnology Information (NCBI), the RNA-seq data for human and avian (chicken and quail) hosts infected with subtypes of influenza A viruses (H1N1, H3N2, H5N1 HALo mutant, H5N1, H5N2), in addition to RNA-seq data for H7N9 infected A549 cells (adenocarcinomic human alveolar basal epithelial cell line) that we generated ourselves. By analyzing the global profiles of differentially expressed genes (DEGs), and the induction of various cytokines and chemokines in human and avian hosts infected with different subtypes of influenza A viruses, we illustrated the differential responses of innate immunity to infections of different subtypes of influenza A viruses in both human and avian hosts. Our results suggest that the viruses which induce stronger innate immune response replicate slower than those induce weaker innate immune response. This work represents a first comprehensive study on innate immune responses in hosts infected with different subtypes of influenza A viruses at an unprecedented scale. It offers new details in the difference of innate immunity to infection of different subtypes of influenza A viruses, and improves our understanding of pathogenesis of influenza A virus and its interaction with the innate immune systems in human and avian hosts.

## Results

### Global profiles of differentially expressed genes in human and avian hosts infected with different subtypes of influenza a viruses

To explore the global changes in gene expression and understand the innate immune responses by hosts infected with different subtypes of influenza A viruses, we first analyzed the RNA-seq data collected from human epithelial cells infected with different subtypes of influenza A viruses, including H1N1, H3N2, H5N1 HALo mutant and H7N9. As noted [26], these were transcriptome data from human bronchial epithelial (HBE) cells infected with H1N1 (PR/8/34) and from human tracheobronchial epithelial (HTBE) cells infected with H1N1 (A/California/04/09), H3N2 (A/Wyoming/03/03) and H5N1 HALo mutant (A/Vietnam/1203/04), respectively (Additional file 1: Table S1). In addition, A549 cells (adenocarcinomic human alveolar basal epithelial cell line) infection experiment with H7N9 (A/Anhui/2013) was performed by our collaborators, for which transcriptome was sequenced (See *Methods*). The result of HBE infected with H1N1(PR/8) is almost the same with that of HTBE infected with H1N1(California/04/09) (See Additional file 2), we use the results of HTBE cells infected with H1N1 as a representative without specifying the result of HBE to make the comparison between different subtypes clearer.

**Fig. 1:**
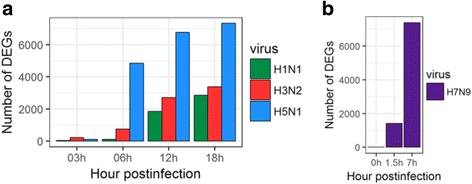
Number of differential expressed genes in HTBE cells infected H1N1, H3N2, H5N1 HALo mutant and A549 cells infected with H7N9. (**a**) Number of differential expressed genes in HTBE cells infected H1N1, H3N2, H5N1 HALo mutant at 03h, 06h, 12h and 24h. (**b**) Number of differential expressed genes in A549 cells infected with H7N9 at 0h, 1.5h and 7h. Number of differentially expressed genes (DEGs) identified from the comparison among mock and virus infected groups (DEGs were identified based on *p*-value threshold of less than 0.05)

**Fig. 2:**
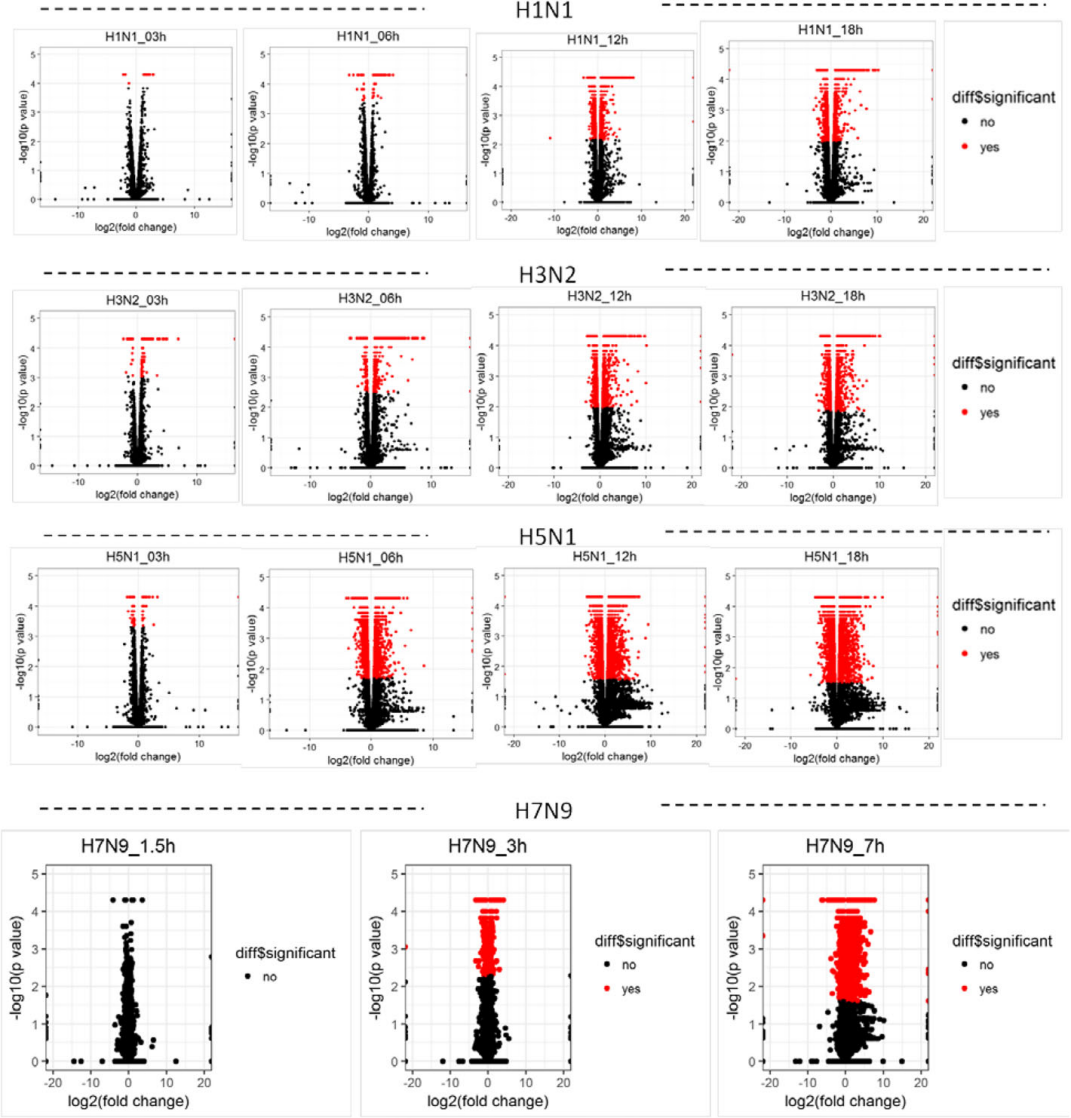
Global overview of DEGs of H1N1, H3N2, H5N1 HALo mutant infected HTBE cells and H7N9 infected A549 cells at different time points. Volcano plot showing DEGs for H1N1, H3N2, H5N1 HALo mutant infected HTBE cells and H7N9 infected A549 cells at different time points. The x-axis represents the log_2_ values of the fold change observed for each mRNA transcript, and the y-axis represents the log_10_ values of the *p*-values of the significance tests between replicates for each transcript. Data for genes that were not classified as differentially expressed are plotted in black

To identify differentially expressed genes (DEGs) at different time points post infection in human hosts infected with different influenza viruses, we used a pipeline similar to what was previously described [24]. Cufflinks software was used to quantify the gene expression and identify the differentially expressed genes (*p*-value <0.05) between control and virus-infected cells. The number of DEGs was highest in H5N1 HALo mutant infected HTBE cells, which was much higher than the that of DEGs in H1N1 and H3N2 infected HTBE cells (Fig. 1a). Timeline-wise, the response to H5N1 HALo mutant infection was abrupt. Similar to H5N1 HALo mutant infected HTBE cells, the number of DEGs in H7N9 infected A549 cells increased abruptly (Fig. 1b), reaching above 6000 at 7 h post infection. The differences in DEG genes and changing dynamics for each influenza subtype are further illustrated in volcano plots, which give more detailed display of the magnitude of changes in contrast to the measure of statistical significance (Fig. 2). To characterize the DEGs infected with different subtypes of influenza A viruses in infected HBE cells or HTBE cells, the DEGs were ranked by fold-change and the top 30 up-regulated DEGs from H1N1, H3N2, or H5N1 HALo mutant infected cells were examined (Additional file 3: Table S2). Gene ontology (GO) enrichment analysis was applied to them (Additional file 4: Table S3, Table S4, Table S5). During early stage of influenza A infection (e.g. 03 h), while no or very few innate immunity-related genes were detected with significant up-regulation in H5N1 HALo mutant or H1N1 infected cells, more up-regulated innate immunity-related genes were detected in H3N2 infected cells (Additional file 4: Table S4), including RIG-I-like receptor dsRNA helicase enzyme (DDX58, IFIH1), CXCL10, CXCL11, chemokine (C-X3-C motif) ligand1 (CX3CL1), chemokine (C-C motif) ligand 5 (CCL5). However, we observed a catch-up of DEGs at later time points (e.g. 06 h, 12 h, 18 h and 24 h post infection) in the category of innate immunity-related genes in H1N1 and H5N1 HALo mutant infected cells. Chemokine pathway-associated genes, such as CXCL-10, CXCL-11, and RIG-I like receptors such as DDX58, IFIH1, were among the most significantly up-regulated genes for all the influenza A subtypes.

**Fig. 3:**
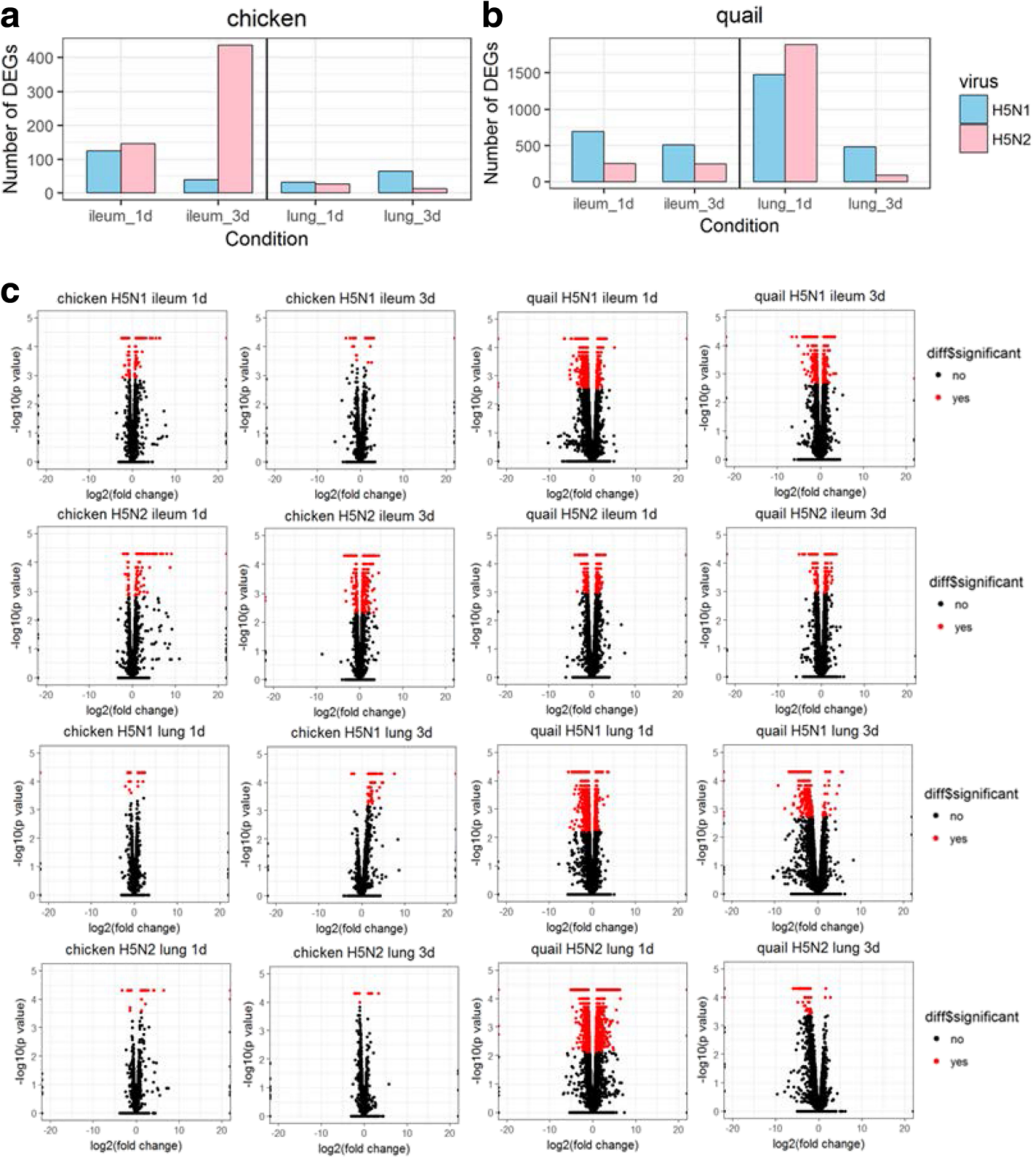
Global overview of DEGs in ileum and lung of chicken and quail infected with H5N1 and H5N2 at different time points. **a-b** Number of differential expressed genes in ileum and lung of chicken and quail infected with H5N1 and H5N2. Number of differentially expressed genes (DEGs) identified from the comparison among mock and virus infected groups (DEGs were identified based on *p*-value threshold of less than 0.05). **c** Volcano plot showing DEGs for ileum and lung of chicken and quail infected with H5N1 and H5N2 at 1 day post infection and 3 days post infection. The x-axis represents the log2 values of the fold change observed for each mRNA transcript, and the y-axis represents the log10 values of the p-values of the significance tests between replicates for each transcript. Data for genes that were not classified as differentially expressed are plotted in black

Second, we further investigated the global changes in gene expression and innate immune responses to infections of influenza A subtypes in Avian, which are possibly the natural hosts of influenza A virus. It has not been clear how the innate immunity in avian hosts responses to different subtypes of influenza A viruses. The transcriptome sequencing data from ileum and lung of chicken and quail infected with H5N1 HALo mutant (A/Vietnam/1203/2004), or H5N2 (A/Mallard/British Columbia/500/2005) were analyzed similarly as described [26]. In chicken, the number of DEGs was significantly higher with H5N2 infection in ileum at 3 day post infection (dpi). In quail, however, the dynamics was quite different. The number of DEGs with H5N1 or H5N2 infection at 1 dpi was much higher in the lung than all of other groups (Fig. 3a, b). They topped out at 1 dpi in the lung, and reduced to near baseline at 3 dpi. The details of changing dynamics and number of significant changes were better illustrated for DEGs with generated volcano plots for the same data sets (Fig. 3c). The distinct response patterns of DEGs by different avian host for different influenza subtypes were revealed for the first time. To understand their possible molecular mechanism, we selected top 10 up-regulated DEGs for further analysis (Additional file 5: Table S6 and Table S7). Surprisingly, different from what was observed in HTBE cells where up-regulated innate immune responsive genes were concentrated, very few innate immunity-related genes were found to be differentially regulated in the lung and ileum of chicken and quail infected with H5N1 and H5N2. Thus, avian, chicken and quail, in general, have a different response pattern of DEGs from that of human hosts when infected with the same subtype, H5N1. In addition, for avian chicken and quail, displayed different responsive pattern of DEGs for infections with the same subtypes of influenza A virus, H5N1, or H5N2.

### Induction of cytokines and chemokines in human lung and tracheobronchial epithelial cells infected with influenza a viruses

**Fig. 4:**
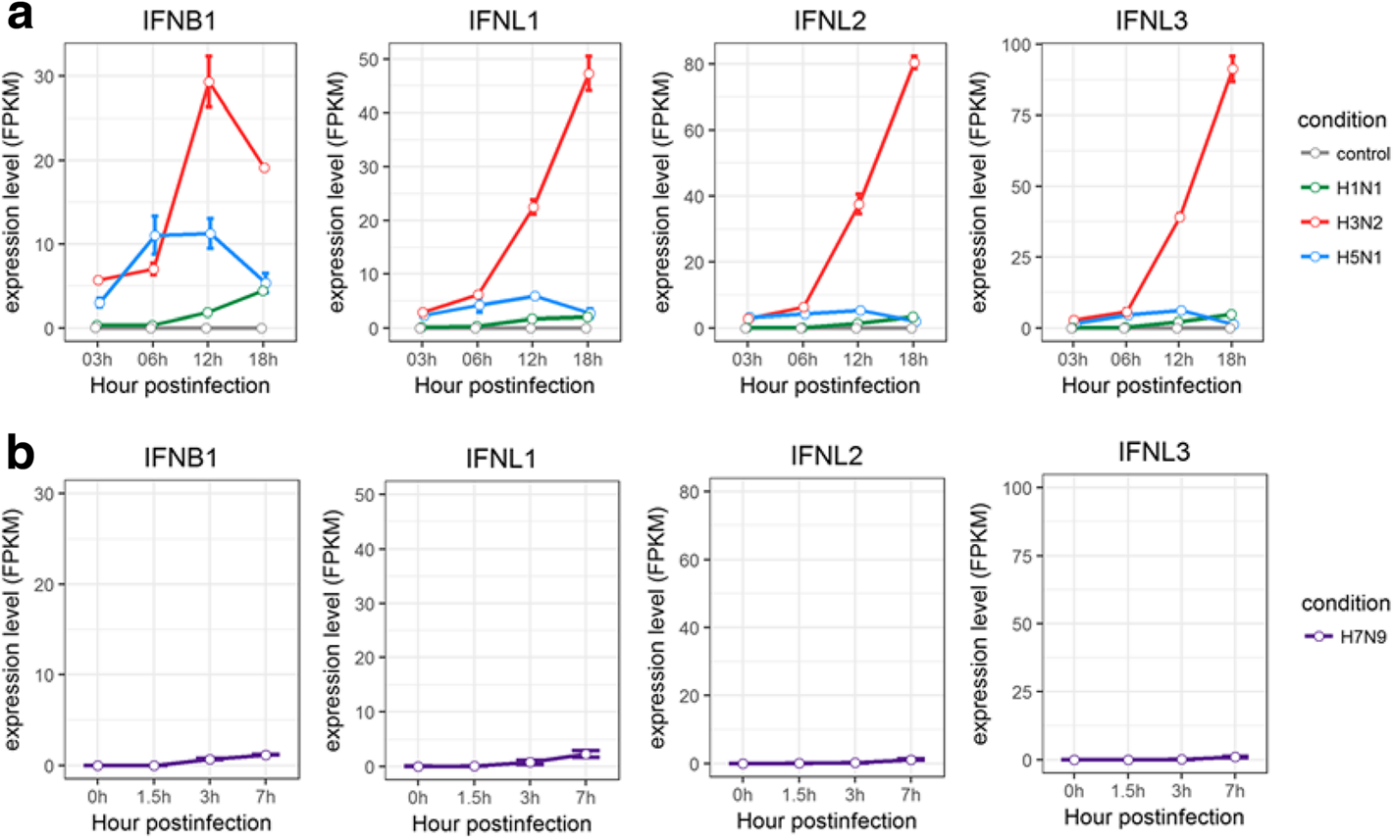
Expression profiles of IFNs in HTBE cells infected with H1N1, H3N2, H5N1 HALo mutant and A549 cells infected with H7N9. **a** The expression levels of IFNs in H1N1, H3N2 and H5N1 HALo mutant infected HTBE cells at different time points. **b** The expression levels of IFNs in H7N9 infected A549 cells at different time points. The gene expression is measured using FPKM reported by Cufflinks (Version: 2.2.1)

To understand the innate immune responses triggered by infections of different subtypes of influenza A viruses, we next focused on induced expression of cytokine and chemokine genes in human and avian hosts infected with either H1N1, H3N2, H5N1 HALo mutant, or H7N9. In HTBE cells infected with H1N1, H3N2, or H5N1 HALo mutant, and A549 cells infected with H7N9, the expression of IFN-β (IFNB1) and IFN-λ (Type III interferon) like IFNL1, IFNL2, and IFNL3 (also called IL29, IL28A, and IL28B respectively), were induced (Fig. 4). While IFN-β was a key signal molecule in the antiviral immune activity [27], Type III interferons comprise a group of newly identified antiviral cytokines that can elicit first-line antiviral responses [28]. Accordingly, H3N2 caused abrupt and the strongest response of IFN-β and IFN-λ, followed by H1N1 though much weaker. On the other hand, H5N1 HALo mutant and H7N9 induced very minor change in expression of type III interferons.

**Fig. 5:**
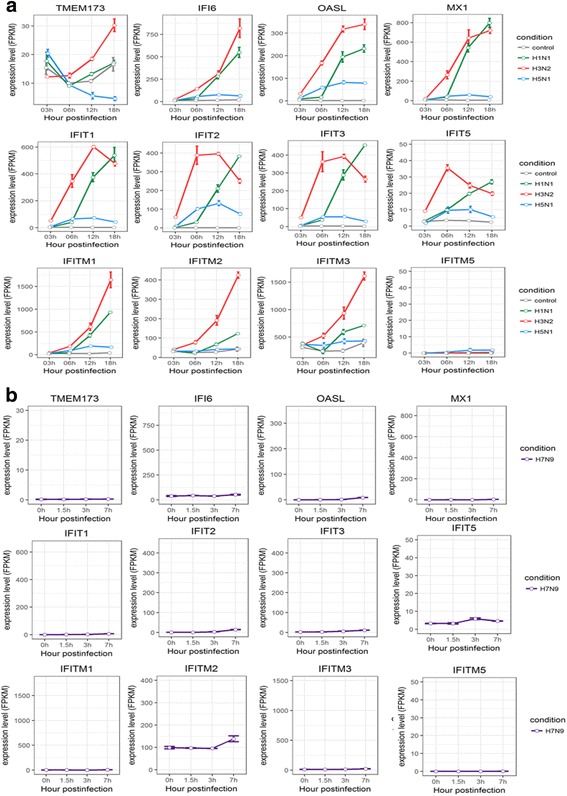
Expression profiles of IFN-stimulated genes in HTBE cells infected with H1N1, H3N2, H5N1 HALo mutant and A549 cells infected with H7N9 at different time points. **a** The expression levels IFN-stimulated genes in H1N1, H3N2 and H5N1 HALo mutant infected HTBE cells at different time points. **b** The expression levels IFN-stimulated genes in H1N1, H3N2 and H5N1 HALo mutant infected HTBE cells at different time points. The gene expression is measured using FPKM reported by Cufflinks (Version: 2.2.1)

TMEM173 (transmembrane protein 173) is known as stimulator of interferon genes (STING) and plays an important role in eliciting  interferon immunity against viral infection [29, 30]. The expression levels of TMEM173 increased during the course of H3N2 infection, deceased with H5N1 HALo mutant infection, and remained constant for both H1N1 and H7N9 (Fig. 5a, b). IFN-stimulated genes (ISGs), such as MX1 (Interferon-induced GTP-binding protein), OASL (oligoadenylate synthetase-like protein), IFI6 (IFNα inducible protein 6), IFITs (The IFN-induced protein with tetratricopeptide repeats) and IFITMs (The interferon inducible transmembrane protein family members), were known as antiviral responder located downstream of the IFN-β and IFN-λ cascades in infected host [31–33]. They were found to be up-regulated to a different extent in cells infected with different subtypes of influenza A viruses (Fig. 5a, b). The H3N2 and H1N1 infected cells had the strongest response, whereas H5N1 HALo mutant and H7N9 had the weakest.

**Fig. 6:**
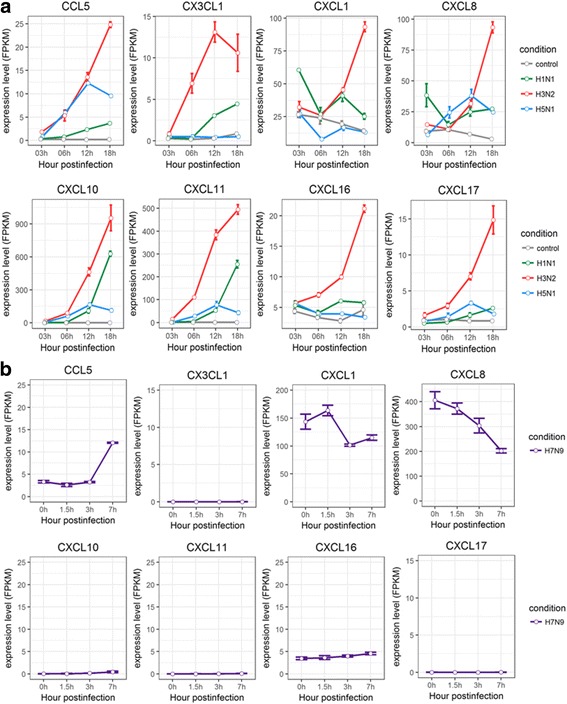
Expression profiles of C-X-C motif ligands, C-C motif ligands in HTBE cells infected with H1N1, H3N2, H5N1 HALo mutant and A549 cells infected with H7N9. **a** The expression levels of CCL5 (C-X-C motif ligand 5), CX3CL1 (C-X3-C motif ligand 1), CXCL1 (C-C motif ligand1), CXCL8, CXCL10, CXCL11, CXCL 16, CXCL17 in H1N1, H3N2, H5N1 HALo mutant infected HTBE cells at different time points. **b** The expression levels of CCL5 (C-X-C motif ligand 5), CX3CL1 (C-X3-C motif ligand 1), CXCL1 (C-C motif ligand1), CXCL8, CXCL10, CXCL11, CXCL 16, CXCL17 in H7N9 infected A549 cells at different time points. The gene expression is measured using FPKM reported by Cufflinks (Version: 2.2.1)

Chemokine (C-C motif) ligand 5 (CCL5), chemokine (C-X3-C motif) ligand 1 (CX3CL1), and chemokine (C-X-C motif) ligands, like CXCL1, CXCL8, CXCL10, CXCL11, CXCL16, and CXCL17, were important pro-inflammatory chemokines [34–37]. They were all induced with significantly higher expression levels for H3N2 and for H5N1 HALo mutant to a less extent. However, their expressions were significant reduced for H5N1 HALo mutant and H7N9 infected cells (Fig. 6a, b).

**Fig. 7:**
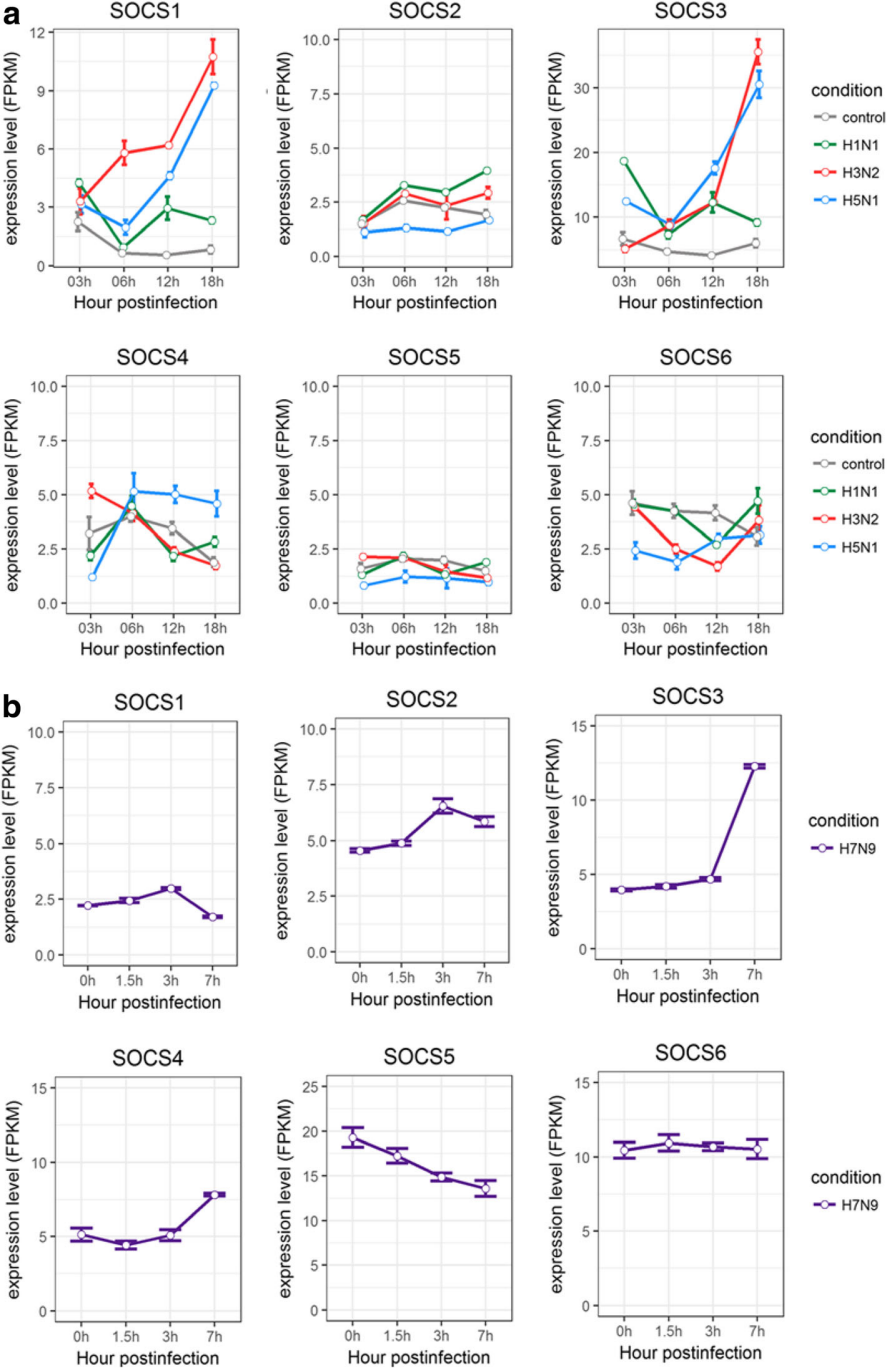
Expression profiles of suppressors of cytokines in HTBE cells infected with H1N1, H3N2, H5N1 HALo mutant and A549 cells infected with H7N9. **a** The expression levels of suppressors of cytokines (SOCS1, SOCS2, SOCS3, SOCS4, SOCS5, SOCS6, SOCS7) in H1N1, H3N2 and H5N1 HALo mutant infected HTBE cells at different time points. **b** The expression levels of suppressors of cytokines (SOCS1, SOCS2, SOCS3, SOCS4, SOCS5, SOCS6, SOCS7) in H7N9 infected A549 cells at different time points. The gene expression is measured using FPKM reported by Cufflinks (Version: 2.2.1)

The SOCS (suppressors of cytokine signaling) molecules were known to negatively regulate inflammatory signaling pathways by facilitating ubiquitination and proteosomal degradation of signaling molecules [38–41]. The expression levels of SOCSs, especially SOCS1 and SOCS3, were found to be up-regulated in H3N2 and H5N1 HALo mutant infected cells (Fig. 7a), while those of SOCS3 and SOCS4 increased significantly in A549 cells infected with H7N9 (Fig. 7b).

Taken together, different patterns of induction for cytokines and chemokines in HTBE cells infected with H1N1, H3N2, H5N1 HALo mutant, or A549 cells infected with H7N9 were observed. In general, H1N1 and H3N2 consistently triggered strong response in production of various antiviral cytokines and chemokines, whereas H5N1 and H7N9 consistently induced weak or no response of these same cytokines and chemokines. Given that H5N1 and H7N9 were reported to be more virulent in comparison with H1N1 and H3N2 [7], these results indicated that virulent strains may be able to avoid triggering strong innate immune response in infected host cells. On the other hand, despite the lower induced expression levels of IFNs for H1N1 infected cells, the expression levels of the ISGs it induced were comparable to that of H3N2 (Fig. 5a), suggesting alternative mechanism other than IFNs may trigger the synthesis of these ISGs. To our surprise, the SOCSs, especially SOCS1 and SOCS3, were significantly up-regulated in H3N2 and H5N1 HALo mutant infected HTBE cells. Given that the H3N2 infection also caused the strongest response in production of various antiviral cytokines and chemokines, the simultaneous up-regulation of SOCS1 and SOCS3 gene expression might be a counter-measurement to limit harmful excessive response. In H5N1 HALo mutant infected cells (which had the highest levels of viral replication; see next session), the up-regulation of SOCS1 and SOCS3 expression might be a mechanism for influenza A viruses to evade host response through inhibiting antiviral cytokine signaling.

### Replication kinetics of different subtypes of influenza a viruses in human lung and tracheobronchial epithelial cells

To understand the pathogenicity or virulence displayed by different subtypes of influenza A viruses, one critical measurement is the replication kinetics of each virus type in infected host cells. It is imperative to examine the relationship between virulence factors and the induced response of the different expression levels of different cytokines and chemokines in human lung and tracheobronchial epithelial cells infected with different subtypes of influenza A viruses.

**Fig. 8:**
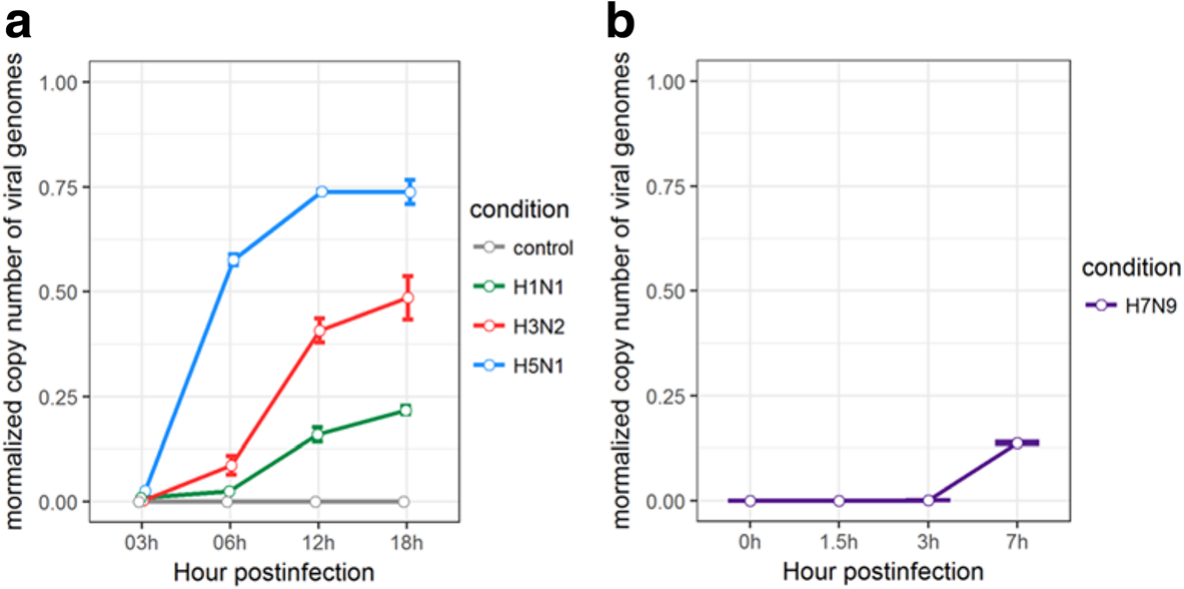
Replication kinetics of H1N1, H3N2, H5N1 HALo mutant in HTBE and H7N9 in A549 cells. **a** Normalized copy number of different influenza A viruses’ genomes in HTBE cells infected with H1N1, H3N2, H5N1 HALo mutant. **b** Normalized copy number of different influenza A viruses’ genomes in A549 cells infected with H7N9. Normalized copy number of genomes is achieved by mapping the clean RNA sequencing reads to the virus reference genome sequences using Burrows-Wheeler algorithm [39], employed by TopHat program (TopHat v2.0.11)

The replication kinetics of H1N1, H3N2, H5N1 HALo mutant and H7N9 subtypes were analyzed by calculating normalized copy number of viral genome in infected cells (Fig. 8). H5N1 HALo mutant was found to have the highest viral replication rate, followed by H3N2, and H1N1. Although the replication rate of H7N9 was not comparable due to different host cell type, A549, it roughly fell in the neighbor of H1N1 or H3N2. Given that H5N1 HALo mutant had the weakest response of induced expression of all examined cytokines and chemokines in infected HTBE cells, these results suggest that the viruses which induce stronger innate immune response replicate slower than those induce weaker innate immune response.

### Induction of expression of innate immune response genes in avian hosts infected with H5N1 and H5N2

Avian are possibly the natural hosts for some subtypes of influenza A virus. It remained unclear how the innate immunity of avian hosts responses to various subtypes of influenza A virus infections. Thus, we focused on the innate immune response genes in avian hosts and investigated their activities during the course of influenza A virus infections. The transcriptome sequencing data from ileum and lung of chicken and quail infected with influenza A virus subtypes H5N1, or H5N2 were analyzed. Unlike the observed activity of cytokine and chemokine genes in human hosts, chicken and quail had dispersed responses from their cytokine and chemokine genes during the course of influenza A viral infections (Additional file 6: Table S8 and Table S9), partly due to the fact that many of the innate immunity genes were not found in the avian genomes. Further both avian host appeared to elicit weak innate immune responses, in comparison to human host.

For example, significant changes of innate immune response genes, i.e. SOCS3, TLR3, IFIH1, IL1RL1, IL18R1, IL17REL, and IL18, were found in the lung of H5N1 infected quail at 1 dpi, whereas at 3 dpi the significantly changed genes were shifted to a different set, including SOCS3, TLR7, TLR4, IL2RB, IL1R2, IL18R1, IL17RA, IL22RA2, IL2RG, IL16, IL21R, IL10RA, and IL7R (Additional file 6: Table S9).

In H5N1 infected chicken, SOCS1 and SOCS3 were up-regulated in the ileum and lung at 3dpi, which was similar to that of SOCS1 and SOCS3 genes in HTBE cells infected with H5N1 (Fig. 7a).These result suggested that H5N1 induced weak innate immune responses in avian hosts that was similar to what was observed in human HTBE cells. It is also interesting to find out the common mechanism in human and avian hosts in which H5N1 infection triggered upregulation of SOCS1 and SOCS3 genes.

Taken together, in comparison with HTBE cells fewer immune-related genes were found to be differentially expressed both in ileum and lung of chicken and quail. The patterns of the differentially expressed immune-related genes were found to be dispersed compared to human hosts. H5N1 caused weaker immune responses in chicken than quail, some of which were similar to what was observed in HTBE cells infected with H5N2.

## Discussion

The innate immune response orchestrated within the hosts following infection with influenza A virus is critical for defense against influenza A virus. The mechanisms underlying this response were not fully understood but seemed to be related to inherent difference in the virus-host interactions [42]. Our study was designed to take advantages of the large collection of transcriptome sequencing data of different hosts infected with different subtypes of influenza A viruses, which were only available recently. We examined the innate immune responses with three variables, different hosts, different subtypes of influenza A viruses, and time-course measurement. Although the data of avian hosts infected with H5N1 and H5N2 was obtained in vivo experiments, the comparison between human and avian host infected with H5N1 may give us useful information to understand the pathogenic mechanism in different hosts. The induced expression patterns of different cytokines and chemokines in infected hosts were revealed with some important observations.

First, the expression levels of IFNs and different antiviral cytokines and chemokines were much lower in H5N1 HALo mutant infected cells, which suggests that the more virulent strain may trigger only weaker antiviral immune response that fail to limit the replications of the influenza viruses. This results were also in accordance with the results of previous studies [10, 11], which reported that the higher virulence of H5N1 correlated with the weaker induction of innate immune responses. It is also interesting to note that the new IFN family, type III interferons, i.e. IFN-λ (IFNL1, IFNL2, IFNL3) [43] were robustly up-regulated in H3N2 infected cells. In contrast with well studied type I interferons known for antiviral activities [44], understanding of the antiviral mechanisms by type III interferons in mucosal surfaces was limited. However, increasing reports suggested a critical role of type III interferons in antiviral response [45]. Our results indicated that the difference between abrupt increase of type III IFN in H3N2 infected cells and the low induction of type III IFN in H1N1 and H5N1 HALo mutant infected cells may be critical factors contributing to their different pathogenicities.

Second, the induced expression of ISGs, such as MX1, OASL, IFI6, IFITs and IFITMs, were much higher in spite of the lower expression of IFNs in H1N1 infected cells. We suggest that there is a complex regulation for synthesis of these ISGs, and the expression of ISGs may provide antiviral defense without the need for inducible IFN production. This observation is in line with results from other studies [46, 47]. Among all the different chemokine (C-X-C motif) ligands, CXCL10 was found to be up-regulated most extensively. Previous studies showed the up-regulation of CXCL10 in several cell types and in response to different pro-inflammatory molecules, always in conjunction with IFN-γ (Type II IFN) [48–50]. However, our results suggest that the increase in the production of CXCL10 may be independent with IFN-γ in HTBE cells during the course of influenza A virus infection.

Third, the suppressors of cytokine signaling, SOCS1 and SOCS3, were significantly up-regulated in H3N2 and H5N1 HALo mutant infected HTBE cells, as well as the lung of chicken infected with H5N1. We suggest the SOCSs might be up-regulated to prevent excessive inflammation in H3N2 infected cells, which is consistent with a recent study showing that excessive expression of type III IFN in lung during influenza A virus infection was followed with a suppression of type III IFN signaling by SOCS-1 [39]. In addition, it is likely the inhibition of host immune response by SOCSs sustained replication of H5N1 virus in human and chicken, which agrees with the result of a recent study [51].

Lastly, in comparison to human hosts, fewer innate immunity genes were found to be differentially expressed in ileum and lung of chicken and quail infected with H5N1 or H5N2. In addition, chicken and quail responded differently to H5N1 or H5N2 infections. Specifically, more DEGs and innate immunity genes were found in quail than in chicken during the course of infection with H5N1. It is worth noting that quail usually survived a few more days than chicken for infection with H5N1 [52], which suggested quail ran a pathological course different from chicken [53]. We further showed that H5N1 caused weaker immune responses in chicken than in quail, and some responsive pattern in chicken was similar to that of HTBE cells. H5N2 triggered stronger immune responses at the early stage in the lung of quail, whereas the innate immune response in the ileum of chicken was more reactive to H5N2. These results demonstrated different pathogenicities of H5N1 and H5N2 viruses in chicken and quail.

In summary, with the extensive data collections from different hosts infected with different subtypes of influenza A viruses, we have systematically characterized the expression patterns of innate immunity-related genes in the hosts, identifying the important difference in innate immune responses of different hosts infected with different subtypes of influenza A viruses. Importantly, we have shown that the failure to elicit strong early innate immune responses was crucial for the pathogenicity of H5N1. We also demonstrated that the expression of particular ISGs may provide antiviral defense without the need for inducible IFN production, and the up-regulation of CXCL10 may be also independent with IFN-γ. The suppressors of cytokine signaling, SOCS1 and SOCS3, may prevent excessive inflammation for H3N2. In general, these results revealed the complex interactions between cytokines, chemokines, PRRs, and the viral factors of different subtypes of influenza A viruses. To our knowledge, this represents the first in depth analysis of the differential innate immune responses in human and avian hosts to infection of different influenza A subtypes, offering new insights into pathogenesis of different subtypes of influenza A viruses and can also guide host-directed antiviral development.

## Conclusion

Our study illustrated the differential responses of innate immunity to infections of different subtypes of influenza A viruses in both human and avian hosts. In general, H1N1 and H3N2 consistently triggered strong response in production of various antiviral cytokines and chemokines, whereas H5N1 and H7N9 infections consistently had weak or no response of these same cytokines and chemokines. Our results suggest that the viruses which induce stronger innate immune response replicate slower than those induce weaker innate immune response. Our study provides important insight into links between the differential innate immune responses from hosts and the pathogenicity/ virulence of different subtypes of influenza A viruses.

## Methods

### Cells and virus

Human alveolar basal epithelial (A549) cells and Madin-Darby canine kidney (MDCK) cells were obtained from the American Type Culture Collection (ATCC, Manassas, VA). A549 and MDCK cells were cultured in Dulbecco’s modified Eagle’s medium (DMEM)(HyClone;South Logan, UT) supplemented with 10% fetal bovine serum (FBS) (Invitrogen Corp., Carlsbad, CA) and 100 U/mL penicillin G sodium and 100 μg/mL streptomycin sulfate(Invitrogen Corp., Carlsbad, CA). Cells were regularly checked for mycoplasma contamination by PCR.

Influenza H7N9 virus strain A/Anhui/1/2013 was obtained from National Institute for Viral Disease Control and Prevention, Chinese Center for Disease Control and Prevention. Virus was propagated in 9–11-day-old specific pathogen free (SPF) embryonated chicken eggs, and viral-infected amnio-allantoic fluid (AAF) was harvested after 3 days post infection. Virus stocks were titrated by standard plaque assay on MDCK cells using an agar overlay medium.

### Virus infection

A549 Cells were washed with PBS and then infected with influenza at the indicated MOIs in infection buffer (DMEM medium containing 2 μg/ml TPCK trypsin and antibiotic: 100 U Penicillin G, 100 μg Streptomycin / ml) for 60 min at 37 °C. Cells were washed again (in infection buffer) and incubated for the indicated time periods at 37 °C in infection buffer. All infection experiments were performed under biosafety level (BSL) 3 conditions.

### Total RNA extraction and RNA sequencing

Total RNA in the virus treated A549 cells were extracted by using RNeasy Mini Kit (Qiagen; Valencia, CA) following the manual instructions. The sequencing library was prepared using Illumina TruSeq RNA sample preparation kit v2 following the manual instructions. The mRNA was derived from total RNA using poly-T oligo-attached magnetic beads and the mRNA was then fragmented and converted into cDNA. The adapters were ligated to the cDNA and the fragments were then amplified by polymerase chain reaction (PCR). We used Illumina Hiseq 2000 to perform paired-end sequencing (101 × 2). All of the RNA-seq data have been deposited in NCBI Gene Expression Omnibus (GEO) under accession code GSE97949.

### RNA sequencing data collection

The main sources of the data used in this study are from the NCBI Sequence Read Archive (SRA; http://www.ncbi.nlm.nih.gov/sra) and NCBI Gene Expression Omnibus (GEO; http://www.ncbi.nlm.nih.gov/geo/). More details on the sequencing data are found in Additional file 1: Table S1. For analysis, the reference genome and gene annotation data for quail are downloaded from "Japanese Quail (*Coturnix japonica*) Genome Sequencing Project" website [54], the reference genome and gene annotation data for human and chicken are downloaded from Illumina’s iGenomes project [55]. The reference genome data for different subtypes of influenza A viruses are downloaded from GISAID (Global Initiative on Sharing All Influenza Data) (http://platform.gisaid.org/epi3/).

### Pipeline for the differential analysis of gene expression

The workflow of this study is shown in Additional file 7: Fig. S1. The raw RNA sequencing data were first processed to remove adapters using Trim galore! [56], and then removed low quality reads with a quality score of 20 using Trimmomatic [57]. Then we evaluated the clean datasets with FastQC [58]. The pipeline for the differential analysis of gene expression were previously described by Trapnell et al. [25]. The clean RNA sequencing reads from different species were mapped to their reference genomes using Burrows-Wheeler algorithm [59], employed by TopHat program (TopHat v2.0.11) [60] with the parameters ‘-G reference.gtf’. The mapping files were professed with Cufflinks (Version: 2.2.1) [61] with options “-g genes.gtf” to estimate the gene expression for different hosts [62]. FPKM (fragments per kilobase of transcript per million mapped reads) values were used to measure the gene expression levels. The differentially expressed genes identified by Cuffdiff using t-test [63]. The clean RNA sequencing reads were also mapped to the genome of influenza A virus, the mapped files were then used to measure the viral transcript counts which were achieved with Samtools (Version: 0.1.19) idxstats program.

### Gene ontology (GO) enrichment analysis

The GO database classifies genes according to the three categories:biological process, cellular component, and molecular function, and predicts the function of the selected genes. To characterize the identified genes from differential expression analysis the GO enrichment analysis were then carried out using the R package clusterProfiler [64].

## Additional files

Additional file 1: **Table S1.** The sources of deep-sequencing transcriptome data of hosts infected with different subtypes of influenza viruses (XLSX 14 kb)

Additional file 2: **Figure S2.** Expression profiles of IFNs in HBE cells infected with H1N1. **Figure S3.** Expression profiles of IFN-stimulated genes in HBE cells infected with H1N1. **Figure S4.** Expression profiles of C-X-C motif ligands, C-C motif ligands in HBE cells infected with H1N1 (DOCX 50 kb)

Additional file 3: **Table S2.** Top 30 up-regulated differentially expressed genes (DEGs) for H1N1, H3N2 and H5N1 infected HTBE cells (DOCX 35 kb)

Additional file 4: **Table S3.** Top 30 Gene ontology (GO) analysis for H1N1 infected HTBE cells. **Table S4.** Top 30 Gene ontology (GO) analysis for H3N2 infected HTBE cells. **Table S5.** Top 30 Gene ontology (GO) analysis for H5N1 infected HTBE cells (DOCX 25 kb)

Additional file 5: **Table S6.** Top 10 up-regulated differentially expressed genes (DEGs) in ileum and lung of chicken infected with H5N1 and H5N2. **Table S7.** Top 10 up-regulated differentially expressed genes (DEGs) in ileum and lung of quail infected with H5N1 and H5N2 (DOCX 27 kb)

Additional file 6: **Table S8.** Differentially expressed immune-related genes in chicken. **Table S9.** Differentially expressed immune-related genes in quail (DOCX 21 kb)

Additional file 7: **Fig. S1.** Work flow of this study (DOCX 837 kb)
