# Supplementary material for: Differential responses of innate immunity triggered by different subtypes of influenza a viruses in human and avian hosts
Source: BMC Med Genomics. 2017 Dec 21;10(Suppl 4):70. doi: 10.1186/s12920-017-0304-z (PMC5763291; doi:10.1186/s12920-017-0304-z)
Supplement: Supplementary file 3 — Table S2. Top 30 up-regulated differentially expressed genes (DEGs) for H1N1, H3N2 and H5N1 infected HTBE cells (DOCX 35 kb) [file 12920_2017_304_MOESM3_ESM.docx]

**Table S2.** Top 30 up-regulated differentially expressed genes (DEGs) for H1N1, H3N2 and H5N1 infected HTBE cells

| **time** | **H1N1** |  |  | **H3N2** |  |  | **H5N1** |  |  |
| --- | --- | --- | --- | --- | --- | --- | --- | --- | --- |
| 03h | gene | log2(fold change) | P value | gene | log2(fold change) | P value | gene | log2(fold change) | P value |
|  | ID2 | 2.93971 | 5.00E-05 | IFIT2 | 6.88137 | 5.00E-05 | IFIT2 | 3.1573 | 5.00E-05 |
|  | OVOL1 | 2.85224 | 5.00E-05 | IFIT1 | 4.98853 | 5.00E-05 | MIR3648 | 2.54714 | 4.00E-04 |
|  | CLDN4 | 2.31282 | 5.00E-05 | IFIT3 | 4.8114 | 5.00E-05 | CXCL10 | 2.37274 | 5.00E-05 |
|  | IL36G | 2.28441 | 5.00E-05 | CXCL10 | 4.42096 | 5.00E-05 | BBC3 | 1.81305 | 5.00E-05 |
|  | EPHB3 | 2.23852 | 5.00E-05 | OASL | 4.34408 | 5.00E-05 | IL6 | 1.73551 | 5.00E-05 |
|  | KRT34 | 2.12715 | 5.00E-05 | CXCL11 | 4.28946 | 5.00E-05 | TNFAIP3 | 1.36794 | 5.00E-05 |
|  | PLAUR | 1.96844 | 5.00E-05 | CMPK2 | 3.72207 | 5.00E-05 | SRRM2 | 1.35424 | 5.00E-05 |
|  | CXCL8 | 1.92639 | 5.00E-05 | RSAD2 | 3.66526 | 5.00E-05 | PMAIP1 | 1.34187 | 5.00E-05 |
|  | NCOA7 | 1.85627 | 5.00E-05 | BATF2 | 3.64936 | 5.00E-05 | ATN1 | 1.27791 | 1.00E-04 |
|  | ID1 | 1.84733 | 5.00E-05 | HERC5 | 3.40768 | 5.00E-05 | OASL | 1.26062 | 5.00E-05 |
|  | FOXA1 | 1.72398 | 5.00E-05 | RND1 | 3.27909 | 0.00085 | HSPG2 | 1.24597 | 5.00E-05 |
|  | ID3 | 1.70651 | 5.00E-05 | IRF1 | 2.64578 | 5.00E-05 | KDM6B | 1.2349 | 5.00E-05 |
|  | HCAR3 | 1.69692 | 5.00E-05 | GBP5 | 2.59256 | 5.00E-05 | FAM65A | 1.23148 | 5.00E-05 |
|  | ELF3 | 1.64774 | 5.00E-05 | DDX58 | 2.58899 | 5.00E-05 | IFIT1 | 1.21726 | 5.00E-05 |
|  | TRAF1 | 1.62407 | 5.00E-05 | CX3CL1 | 2.4276 | 5.00E-05 | KMT2D | 1.16721 | 5.00E-05 |
|  | LIF | 1.54302 | 5.00E-05 | ATF3 | 2.3053 | 5.00E-05 | SON | 1.15592 | 5.00E-05 |
|  | TICAM1 | 1.53667 | 5.00E-05 | FAM46A | 2.25711 | 5.00E-05 | IFIT3 | 1.13143 | 5.00E-05 |
|  | BCL2A1 | 1.52133 | 5.00E-05 | IFIH1 | 2.22797 | 5.00E-05 | ATF3 | 1.13036 | 5.00E-05 |
|  | SERPINB2 | 1.44953 | 5.00E-05 | MX2 | 2.19113 | 5.00E-05 | EP400 | 1.11102 | 5.00E-05 |
|  | THEMIS2 | 1.44133 | 5.00E-05 | GBP4 | 2.17 | 5.00E-05 | ZMIZ1 | 1.11096 | 5.00E-05 |
|  | KRT75 | 1.43747 | 5.00E-05 | HSH2D | 2.15974 | 5.00E-05 | PTGER4 | 1.07678 | 5.00E-05 |
|  | SEMA7A | 1.42485 | 5.00E-05 | ISG15 | 2.07122 | 5.00E-05 | LRP1 | 1.0755 | 5.00E-05 |
|  | SOCS3 | 1.40532 | 5.00E-05 | OAS1 | 2.06938 | 5.00E-05 | EGR1 | 1.0661 | 5.00E-05 |
|  | ITPKC | 1.35348 | 5.00E-05 | CCL5 | 2.05751 | 5.00E-05 | PTPN23 | 1.04007 | 5.00E-05 |
|  | CXCL1 | 1.33338 | 5.00E-05 | IFI44L | 2.04964 | 5.00E-05 | POLR2A | 1.03512 | 5.00E-05 |
|  | TMCC3 | 1.33273 | 5.00E-05 | PMAIP1 | 1.94293 | 5.00E-05 | EP300 | 1.0175 | 5.00E-05 |
|  | FOS | 1.32525 | 5.00E-05 | HELZ2 | 1.93721 | 5.00E-05 | COL12A1 | 0.996821 | 5.00E-05 |
|  | AP5B1 | 1.32342 | 5.00E-05 | MX1 | 1.8159 | 5.00E-05 | ARID1A | 0.986502 | 5.00E-05 |
|  | CDC42EP1 | 1.27524 | 5.00E-05 | IFI44 | 1.79285 | 5.00E-05 | KMT2A | 0.983532 | 5.00E-05 |
|  | TSC22D1 | -1.24071 | 1.00E-04 | XAF1 | 1.78152 | 5.00E-05 | SETD1B | 0.959677 | 0.00025 |
| 06h | IFIT2 | 4.13386 | 5.00E-05 | IFIT2 | 8.84019 | 5.00E-05 | CCDC169 | 8.55536 | 0.0079 |
|  | IFIT1 | 3.57831 | 5.00E-05 | CMPK2 | 8.62692 | 5.00E-05 | NUP210L | 6.44588 | 0.00055 |
|  | IFIT3 | 3.462 | 5.00E-05 | IFIT1 | 8.61764 | 5.00E-05 | IFIT2 | 5.85441 | 5.00E-05 |
|  | CMPK2 | 3.26853 | 5.00E-05 | RSAD2 | 8.55532 | 5.00E-05 | AGBL2 | 5.51452 | 0.00175 |
|  | RSAD2 | 2.8883 | 5.00E-05 | IFIT3 | 7.77849 | 5.00E-05 | SLC16A12 | 5.4652 | 0.01495 |
|  | CXCL11 | 2.83843 | 5.00E-05 | CXCL11 | 7.5946 | 5.00E-05 | CXCL11 | 5.22528 | 5.00E-05 |
|  | OASL | 2.61582 | 5.00E-05 | IFI44L | 7.48986 | 5.00E-05 | CXCL10 | 5.21864 | 5.00E-05 |
|  | BATF2 | 2.45934 | 5.00E-05 | CXCL10 | 7.36396 | 5.00E-05 | TRIM69 | 5.1469 | 0.0094 |
|  | HSH2D | 2.39986 | 5.00E-05 | OASL | 7.26955 | 5.00E-05 | COLEC11 | 4.80659 | 0.00015 |
|  | DDX58 | 2.18402 | 5.00E-05 | MX2 | 7.09835 | 5.00E-05 | CMPK2 | 4.59258 | 5.00E-05 |
|  | OAS1 | 2.17599 | 5.00E-05 | ETV7 | 6.93628 | 0.00025 | HSPA6 | 4.53415 | 5.00E-05 |
|  | HSPA7 | 2.06651 | 3.00E-04 | BATF2 | 6.92505 | 5.00E-05 | OASL | 4.5032 | 5.00E-05 |
|  | CXCL10 | 2.05289 | 5.00E-05 | MX1 | 6.29346 | 5.00E-05 | RNF144A-AS1 | 4.43497 | 0.0061 |
|  | MX1 | 2.03388 | 5.00E-05 | HSH2D | 6.21974 | 5.00E-05 | CCL5 | 4.29772 | 5.00E-05 |
|  | GBP5 | 2.01282 | 5.00E-05 | RTP4 | 6.19367 | 0.00125 | NCOA7 | 4.29209 | 5.00E-05 |
|  | IFIH1 | 1.89712 | 5.00E-05 | GBP4 | 5.77791 | 5.00E-05 | IFIT1 | 4.2558 | 5.00E-05 |
|  | IFI44L | 1.88265 | 5.00E-05 | HERC5 | 5.65586 | 5.00E-05 | HERC5 | 4.16862 | 5.00E-05 |
|  | HERC5 | 1.78773 | 5.00E-05 | GBP5 | 5.64969 | 5.00E-05 | IL16 | 4.07067 | 5.00E-05 |
|  | OCLN | 1.65817 | 5.00E-05 | OAS1 | 5.57551 | 5.00E-05 | CCDC62 | 3.99584 | 6.00E-04 |
|  | ISG15 | 1.62715 | 5.00E-05 | ISG15 | 5.36822 | 5.00E-05 | LINC00869 | 3.94342 | 0.01125 |
|  | GBP4 | 1.58129 | 0.00025 | SAMD9L | 5.19328 | 5.00E-05 | IFIT3 | 3.92336 | 5.00E-05 |
|  | KRT34 | 1.57994 | 5.00E-05 | XAF1 | 5.16868 | 5.00E-05 | GBP4 | 3.87856 | 5.00E-05 |
|  | IFI44 | 1.56528 | 5.00E-05 | CX3CL1 | 5.12562 | 5.00E-05 | CCDC81 | 3.83009 | 0.00655 |
|  | MX2 | 1.47158 | 5.00E-05 | APOL4 | 5.03134 | 2.00E-04 | GBP5 | 3.8082 | 5.00E-05 |
|  | USP18 | 1.45861 | 5.00E-05 | IFIH1 | 4.97257 | 5.00E-05 | HSPA7 | 3.7573 | 0.00605 |
|  | OAS2 | 1.4519 | 5.00E-05 | DDX58 | 4.91539 | 5.00E-05 | IFI44L | 3.61664 | 5.00E-05 |
|  | IFIT5 | 1.43992 | 5.00E-05 | LAMP3 | 4.83346 | 5.00E-05 | LOC100130705 | 3.603 | 0.00295 |
|  | HELZ2 | 1.39505 | 5.00E-05 | DDX60L | 4.70783 | 5.00E-05 | SYNPO2 | 3.59703 | 1.00E-04 |
|  | IFI6 | 1.31037 | 5.00E-05 | IFI44 | 4.56576 | 5.00E-05 | KIAA1549 | 3.58737 | 5.00E-05 |
|  | SAMD9L | 1.27695 | 5.00E-05 | THEMIS2 | 4.3973 | 5.00E-05 | PPP1R36 | 3.47002 | 0.01485 |
| 12h | CMPK2 | 8.09034 | 5.00E-05 | CXCL10 | 10.1525 | 1.00E-04 | ID2 | 9.44268 | 4.00E-04 |
|  | CXCL10 | 7.89762 | 5.00E-05 | CMPK2 | 10.0572 | 0.00165 | CXCL10 | 8.85965 | 0.00115 |
|  | RSAD2 | 7.48794 | 5.00E-05 | RSAD2 | 9.81128 | 5.00E-05 | HSPA6 | 7.40507 | 5.00E-05 |
|  | IFIT1 | 7.33667 | 5.00E-05 | CXCL11 | 9.70602 | 5.00E-05 | FOSB | 7.34051 | 0.0033 |
|  | IFIT2 | 7.24976 | 5.00E-05 | IFIT1 | 8.88408 | 5.00E-05 | AGBL2 | 7.21317 | 0.00515 |
|  | CXCL11 | 7.07714 | 5.00E-05 | IFIT2 | 8.81228 | 5.00E-05 | CXCL11 | 7.14127 | 5.00E-05 |
|  | MX2 | 7.04469 | 5.00E-05 | MX2 | 8.62445 | 5.00E-05 | RASD1 | 7.07175 | 0.0197 |
|  | IFIT3 | 6.61329 | 5.00E-05 | ETV7 | 8.28401 | 7.00E-04 | RNF144A-AS1 | 6.61661 | 0.01825 |
|  | MX1 | 6.60464 | 5.00E-05 | IFI44L | 8.23066 | 5.00E-05 | NCOA7 | 6.60994 | 5.00E-05 |
|  | OASL | 6.54075 | 5.00E-05 | OASL | 8.09176 | 5.00E-05 | IFIT2 | 6.59188 | 5.00E-05 |
|  | GMPR | 6.21159 | 5.00E-05 | GMPR | 8.05665 | 0.0065 | RND1 | 6.26113 | 5.00E-05 |
|  | ETV7 | 6.17031 | 5.00E-05 | MX1 | 7.6096 | 5.00E-05 | FAM46C | 6.22022 | 0.02645 |
|  | BATF2 | 6.02984 | 5.00E-05 | IFIT3 | 7.43878 | 5.00E-05 | RSAD2 | 6.08109 | 5.00E-05 |
|  | IFI44L | 5.93274 | 5.00E-05 | HERC5 | 7.00811 | 5.00E-05 | FBXW10 | 6.0329 | 0.02545 |
|  | HERC5 | 5.59229 | 5.00E-05 | GBP4 | 6.87002 | 5.00E-05 | CCDC62 | 5.84758 | 0.0012 |
|  | HSPA7 | 5.51748 | 5.00E-05 | RTP4 | 6.83038 | 0.00315 | CMPK2 | 5.79525 | 5.00E-05 |
|  | HSH2D | 5.2693 | 5.00E-05 | ATP6V0A4 | 6.80595 | 0.00865 | HERC5 | 5.6412 | 5.00E-05 |
|  | OAS1 | 5.25752 | 5.00E-05 | GBP5 | 6.7788 | 5.00E-05 | ITGA7 | 5.40003 | 0.0257 |
|  | ISG15 | 5.21269 | 5.00E-05 | HSH2D | 6.58951 | 5.00E-05 | IKZF3 | 5.34114 | 5.00E-05 |
|  | GBP4 | 5.19316 | 5.00E-05 | BATF2 | 6.58887 | 5.00E-05 | GBP4 | 5.30235 | 5.00E-05 |
|  | RASGRP3 | 5.09046 | 5.00E-05 | OAS1 | 6.37105 | 5.00E-05 | FER1L6 | 5.2908 | 0.0087 |
|  | FAR2P2 | 4.97616 | 0.00295 | ISG15 | 6.31779 | 5.00E-05 | OASL | 5.27743 | 5.00E-05 |
|  | LGALS9 | 4.92863 | 5.00E-05 | APOL3 | 6.05102 | 4.00E-04 | LINC00869 | 5.27064 | 0.01355 |
|  | LAMP3 | 4.8818 | 5.00E-05 | MMP13 | 6.04255 | 0.00135 | SLC16A12 | 5.26292 | 0.02645 |
|  | RTP4 | 4.81484 | 5.00E-05 | APOL4 | 5.9125 | 0.00395 | CCL5 | 5.26221 | 5.00E-05 |
|  | MTRNR2L1 | 4.78724 | 5.00E-05 | XAF1 | 5.64737 | 5.00E-05 | FAM71F2 | 5.23916 | 0.0079 |
|  | MMP13 | 4.6576 | 5.00E-05 | HSPA6 | 5.59474 | 5.00E-05 | TG | 5.22887 | 0.00495 |
|  | NUP210L | 4.60969 | 5.00E-05 | SECTM1 | 5.5743 | 5.00E-05 | RNU12 | 5.22763 | 5.00E-05 |
|  | USP18 | 4.58255 | 5.00E-05 | LAMP3 | 5.41184 | 5.00E-05 | CMYA5 | 5.17346 | 0.00675 |
|  | GBP5 | 4.53807 | 5.00E-05 | IFI44 | 5.39969 | 5.00E-05 | CDRT1 | 5.15921 | 2.00E-04 |
| 18h | CXCL10 | 10.249 | 5.00E-05 | CXCL10 | 10.0968 | 5.00E-05 | ID2 | 9.87226 | 5.00E-05 |
|  | CXCL11 | 9.6279 | 5.00E-05 | CXCL11 | 9.99006 | 5.00E-05 | HSPA6 | 8.3151 | 5.00E-05 |
|  | RSAD2 | 9.33628 | 5.00E-05 | RSAD2 | 9.84364 | 5.00E-05 | RND1 | 7.8589 | 0.00895 |
|  | CMPK2 | 9.10147 | 5.00E-05 | MX2 | 9.06746 | 5.00E-05 | AGBL2 | 7.63807 | 0.0213 |
|  | IFIT2 | 8.09216 | 5.00E-05 | IFIT2 | 8.59142 | 5.00E-05 | RGS16 | 7.5019 | 0.02125 |
|  | MX2 | 7.76675 | 5.00E-05 | IFI44L | 8.51665 | 5.00E-05 | CXCL10 | 7.24591 | 1.00E-04 |
|  | IFIT1 | 7.66096 | 5.00E-05 | IFIT1 | 7.98259 | 5.00E-05 | CXCL11 | 6.92009 | 0.0076 |
|  | ZBP1 | 7.64137 | 9.00E-04 | GBP4 | 7.81445 | 5.00E-05 | RNF144A-AS1 | 6.77493 | 0.0239 |
|  | IFIT3 | 7.17741 | 5.00E-05 | LGALS9 | 7.79277 | 5.00E-05 | CCDC169 | 6.75304 | 0.00615 |
|  | HERC5 | 7.04115 | 5.00E-05 | OASL | 7.63563 | 5.00E-05 | NCOA7 | 6.69381 | 5.00E-05 |
|  | IFI44L | 6.91547 | 5.00E-05 | MX1 | 7.53664 | 5.00E-05 | FAM46C | 6.68401 | 0.02525 |
|  | GMPR | 6.90417 | 5.00E-05 | LAMP3 | 7.48055 | 5.00E-05 | IKZF3 | 6.45391 | 5.00E-05 |
|  | LAMP3 | 6.85869 | 5.00E-05 | HERC5 | 7.44416 | 5.00E-05 | RSAD2 | 6.32041 | 1.00E-04 |
|  | GBP4 | 6.77438 | 5.00E-05 | IFIT3 | 7.35304 | 5.00E-05 | RNU12 | 6.18182 | 5.00E-05 |
|  | MX1 | 6.63524 | 5.00E-05 | HSPA6 | 7.28108 | 5.00E-05 | IFIT2 | 6.00116 | 5.00E-05 |
|  | GBP5 | 6.63274 | 5.00E-05 | MMP13 | 7.25315 | 0.0014 | CDRT1 | 5.89752 | 0.00375 |
|  | LGALS9 | 6.57289 | 5.00E-05 | IFI44 | 7.11017 | 5.00E-05 | BCO2 | 5.88945 | 0.00525 |
|  | RASGRP3 | 6.55964 | 7.00E-04 | XAF1 | 6.79031 | 1.00E-04 | FAM69B | 5.86178 | 0.00045 |
|  | MMP13 | 6.49203 | 5.00E-05 | OAS1 | 6.77649 | 5.00E-05 | CCDC62 | 5.79065 | 0.00055 |
|  | ETV7 | 6.47895 | 5.00E-05 | BATF2 | 6.62715 | 5.00E-05 | CRISPLD2 | 5.77497 | 0.00755 |
|  | OASL | 6.43512 | 5.00E-05 | ISG15 | 6.62277 | 5.00E-05 | CMPK2 | 5.76262 | 4.00E-04 |
|  | TNFSF13B | 6.32688 | 0.00025 | TNFSF13B | 6.60076 | 0.01295 | PCDH9 | 5.48711 | 2.00E-04 |
|  | AFP | 6.20217 | 5.00E-05 | BST2 | 6.56148 | 5.00E-05 | KLRC1 | 5.47505 | 0.03035 |
|  | BST2 | 6.14733 | 5.00E-05 | FLJ35934 | 6.40258 | 0.00205 | GBP4 | 5.45611 | 0.002 |
|  | HSH2D | 5.89752 | 5.00E-05 | HSH2D | 6.24107 | 5.00E-05 | CCL5 | 5.39835 | 2.00E-04 |
|  | XAF1 | 5.85685 | 5.00E-05 | DDX58 | 6.12393 | 5.00E-05 | TG | 5.29738 | 0.0201 |
|  | BCL2L14 | 5.77985 | 0.00825 | SAMD9L | 6.11843 | 5.00E-05 | CORIN | 5.28472 | 0.00905 |
|  | IFI44 | 5.65501 | 5.00E-05 | GBP5 | 6.00978 | 5.00E-05 | NID1 | 5.27605 | 0.00035 |
|  | OAS1 | 5.64102 | 5.00E-05 | CCL5 | 5.95154 | 5.00E-05 | MAP3K8 | 5.25968 | 5.00E-05 |
|  | SAMD9L | 5.53784 | 5.00E-05 | IFI6 | 5.93078 | 5.00E-05 | KIAA1549 | 5.25842 | 5.00E-05 |
| 24h | RSAD2 | 8.23122 | 5.00E-05 |  |  |  |  |  |  |
|  | CXCL10 | 8.08799 | 5.00E-05 |  |  |  |  |  |  |
|  | ZBP1 | 7.8765 | 0.0025 |  |  |  |  |  |  |
|  | LGALS9 | 7.85754 | 1.00E-04 |  |  |  |  |  |  |
|  | CMPK2 | 7.76381 | 5.00E-05 |  |  |  |  |  |  |
|  | CXCL11 | 7.36737 | 5.00E-05 |  |  |  |  |  |  |
|  | MX2 | 7.29758 | 5.00E-05 |  |  |  |  |  |  |
|  | IFI44L | 6.94559 | 5.00E-05 |  |  |  |  |  |  |
|  | BST2 | 6.64553 | 5.00E-05 |  |  |  |  |  |  |
|  | GBP4 | 6.52957 | 5.00E-05 |  |  |  |  |  |  |
|  | LAMP3 | 6.4809 | 5.00E-05 |  |  |  |  |  |  |
|  | IFIT1 | 6.32615 | 5.00E-05 |  |  |  |  |  |  |
|  | GMPR | 6.29989 | 5.00E-05 |  |  |  |  |  |  |
|  | IFIT2 | 6.29032 | 5.00E-05 |  |  |  |  |  |  |
|  | MX1 | 6.15002 | 5.00E-05 |  |  |  |  |  |  |
|  | MMP13 | 6.14861 | 5.00E-05 |  |  |  |  |  |  |
|  | FLJ35934 | 6.14095 | 0.00205 |  |  |  |  |  |  |
|  | IFIT3 | 5.93347 | 5.00E-05 |  |  |  |  |  |  |
|  | HERC5 | 5.90863 | 5.00E-05 |  |  |  |  |  |  |
|  | ISG15 | 5.86353 | 5.00E-05 |  |  |  |  |  |  |
|  | OASL | 5.79145 | 5.00E-05 |  |  |  |  |  |  |
|  | GBP5 | 5.57794 | 5.00E-05 |  |  |  |  |  |  |
|  | RASGRP3 | 5.5644 | 1.00E-04 |  |  |  |  |  |  |
|  | HSH2D | 5.48512 | 5.00E-05 |  |  |  |  |  |  |
|  | OAS1 | 5.46848 | 5.00E-05 |  |  |  |  |  |  |
|  | ETV7 | 5.37681 | 5.00E-05 |  |  |  |  |  |  |
|  | TNFSF13B | 5.29001 | 5.00E-05 |  |  |  |  |  |  |
|  | IFI44 | 5.25983 | 5.00E-05 |  |  |  |  |  |  |
|  | XAF1 | 5.25657 | 5.00E-05 |  |  |  |  |  |  |
|  | OAS2 | 5.16537 | 5.00E-05 |  |  |  |  |  |  |
