# Supplementary material for: Differential responses of innate immunity triggered by different subtypes of influenza a viruses in human and avian hosts
Source: BMC Med Genomics. 2017 Dec 21;10(Suppl 4):70. doi: 10.1186/s12920-017-0304-z (PMC5763291; doi:10.1186/s12920-017-0304-z)
Supplement: Supplementary file 4 — Table S3. Top 30 Gene ontology (GO) analysis for H1N1 infected HTBE cells. Table S4. Top 30 Gene ontology (GO) analysis for H3N2 infected HTBE cells. Table S5. Top 30 Gene ontology (GO) analysis for H5N1 infected HTBE cells (DOCX 25 kb) [file 12920_2017_304_MOESM4_ESM.docx]

**Table S3** Top 30 Gene ontology (GO) analysis for H1N1 infected HTBE cells

|  | H1N1 | ID | Description | p.adjust | Count |
| --- | --- | --- | --- | --- | --- |
| 03h | IL36G/CXCL8/TRAF1/LIF/CXCL1 | GO:0005126 | cytokine receptor binding | 0.006409 | 5 |
|  | CXCL8/CXCL1 | GO:0045236 | CXCR chemokine receptor binding | 0.013131 | 2 |
|  | IL36G/CXCL8/LIF/CXCL1 | GO:0005125 | cytokine activity | 0.013131 | 4 |
|  | OVOL1/FOXA1/ELF3/FOS | GO:0000982 | transcription factor activity, RNA polymerase II core promoter proximal region sequence-specific binding | 0.047024 | 4 |
|  | CXCL8/CXCL1 | GO:0008009 | chemokine activity | 0.047024 | 2 |
| 06h | OASL/DDX58/OAS1/IFIH1/OAS2 | GO:0003725 | double-stranded RNA binding | 3.28E-06 | 5 |
|  | DDX58/IFIH1/IFIT5 | GO:0003727 | single-stranded RNA binding | 0.004352 | 3 |
|  | CXCL11/CXCL10 | GO:0045236 | CXCR chemokine receptor binding | 0.004352 | 2 |
|  | MX1/GBP5/IFI44L/GBP4/MX2 | GO:0005525 | GTP binding | 0.004352 | 5 |
|  | MX1/GBP5/IFI44L/GBP4/MX2 | GO:0032561 | guanyl ribonucleotide binding | 0.004352 | 5 |
|  | MX1/GBP5/IFI44L/GBP4/MX2 | GO:0019001 | guanyl nucleotide binding | 0.004352 | 5 |
|  | MX1/GBP5/GBP4/MX2 | GO:0003924 | GTPase activity | 0.004579 | 4 |
|  | OAS1/OAS2 | GO:0070566 | adenylyltransferase activity | 0.004659 | 2 |
|  | DDX58/IFIH1/HELZ2 | GO:0004386 | helicase activity | 0.012429 | 3 |
|  | CXCL11/CXCL10 | GO:0008009 | chemokine activity | 0.016184 | 2 |
|  | CXCL11/CXCL10 | GO:0042379 | chemokine receptor binding | 0.022794 | 2 |
| 12h | MX2/MX1/IFI44L/GBP4/GBP5 | GO:0005525 | GTP binding | 0.005918 | 5 |
|  | CXCL10/CXCL11 | GO:0045236 | CXCR chemokine receptor binding | 0.005918 | 2 |
|  | MX2/MX1/IFI44L/GBP4/GBP5 | GO:0032561 | guanyl ribonucleotide binding | 0.005918 | 5 |
|  | MX2/MX1/IFI44L/GBP4/GBP5 | GO:0019001 | guanyl nucleotide binding | 0.005918 | 5 |
|  | MX2/MX1/GBP4/GBP5 | GO:0003924 | GTPase activity | 0.006229 | 4 |
|  | CXCL10/CXCL11 | GO:0008009 | chemokine activity | 0.030305 | 2 |
|  | CXCL10/CXCL11 | GO:0042379 | chemokine receptor binding | 0.037585 | 2 |
|  | OASL/OAS1 | GO:0003725 | double-stranded RNA binding | 0.037585 | 2 |
| 18h | MX2/IFI44L/GBP4/MX1/GBP5 | GO:0005525 | GTP binding | 0.007534 | 5 |
|  | CXCL10/CXCL11 | GO:0045236 | CXCR chemokine receptor binding | 0.007534 | 2 |
|  | MX2/IFI44L/GBP4/MX1/GBP5 | GO:0032561 | guanyl ribonucleotide binding | 0.007534 | 5 |
|  | MX2/IFI44L/GBP4/MX1/GBP5 | GO:0019001 | guanyl nucleotide binding | 0.007534 | 5 |
|  | MX2/GBP4/MX1/GBP5 | GO:0003924 | GTPase activity | 0.007652 | 4 |
|  | CXCL10/CXCL11 | GO:0008009 | chemokine activity | 0.034562 | 2 |
|  | CXCL10/CXCL11 | GO:0042379 | chemokine receptor binding | 0.04284 | 2 |
|  | OASL/OAS1 | GO:0003725 | double-stranded RNA binding | 0.04284 | 2 |
|  | CXCL10/CXCL11/TNFSF13B | GO:0005125 | cytokine activity | 0.045242 | 3 |
| 24h | OASL/OAS1/OAS2 | GO:0003725 | double-stranded RNA binding | 0.006027 | 3 |
|  | MX2/IFI44L/GBP4/MX1/GBP5 | GO:0005525 | GTP binding | 0.006027 | 5 |
|  | CXCL10/CXCL11 | GO:0045236 | CXCR chemokine receptor binding | 0.006027 | 2 |
|  | MX2/IFI44L/GBP4/MX1/GBP5 | GO:0032561 | guanyl ribonucleotide binding | 0.006027 | 5 |
|  | MX2/IFI44L/GBP4/MX1/GBP5 | GO:0019001 | guanyl nucleotide binding | 0.006027 | 5 |
|  | MX2/GBP4/MX1/GBP5 | GO:0003924 | GTPase activity | 0.006376 | 4 |
|  | OAS1/OAS2 | GO:0070566 | adenylyltransferase activity | 0.006816 | 2 |
|  | CXCL10/CXCL11 | GO:0008009 | chemokine activity | 0.025922 | 2 |
|  | CXCL10/CXCL11 | GO:0042379 | chemokine receptor binding | 0.035716 | 2 |
|  | CXCL10/CXCL11/TNFSF13B | GO:0005125 | cytokine activity | 0.040718 | 3 |

**Table S4** Top 30 Gene ontology (GO) analysis for H3N2 infected HTBE cells

|  | H3N2 | ID | Description | p.adjust | Count |
| --- | --- | --- | --- | --- | --- |
| 03h | CXCL10/CXCL11/CX3CL1/CCL5 | GO:0008009 | chemokine activity | 0.0001 | 4 |
|  | CXCL10/CXCL11/CX3CL1/CCL5 | GO:0042379 | chemokine receptor binding | 0.0001 | 4 |
|  | OASL/DDX58/IFIH1/OAS1 | GO:0003725 | double-stranded RNA binding | 0.0001 | 4 |
|  | RND1/GBP5/MX2/GBP4/IFI44L/MX1 | GO:0005525 | GTP binding | 0.000575 | 6 |
|  | RND1/GBP5/MX2/GBP4/MX1 | GO:0003924 | GTPase activity | 0.000575 | 5 |
|  | RND1/GBP5/MX2/GBP4/IFI44L/MX1 | GO:0032561 | guanyl ribonucleotide binding | 0.000575 | 6 |
|  | RND1/GBP5/MX2/GBP4/IFI44L/MX1 | GO:0019001 | guanyl nucleotide binding | 0.000575 | 6 |
|  | CXCL10/CXCL11 | GO:0045236 | CXCR chemokine receptor binding | 0.003676 | 2 |
|  | CXCL10/CXCL11/CX3CL1/CCL5 | GO:0005125 | cytokine activity | 0.004698 | 4 |
|  | CXCL10/CXCL11/CX3CL1/CCL5 | GO:0001664 | G-protein coupled receptor binding | 0.007446 | 4 |
|  | CXCL10/CXCL11/CX3CL1/CCL5 | GO:0005126 | cytokine receptor binding | 0.00802 | 4 |
|  | CX3CL1/CCL5 | GO:0048020 | CCR chemokine receptor binding | 0.011738 | 2 |
|  | DDX58/IFIH1/HELZ2 | GO:0004386 | helicase activity | 0.01295 | 3 |
|  | RSAD2/CCL5 | GO:0043621 | protein self-association | 0.014825 | 2 |
|  | DDX58/IFIH1 | GO:0003727 | single-stranded RNA binding | 0.032838 | 2 |
| 06h | OASL/OAS1/IFIH1/DDX58 | GO:0003725 | double-stranded RNA binding | 0.00013 | 4 |
|  | CXCL11/CXCL10/CX3CL1 | GO:0008009 | chemokine activity | 0.001453 | 3 |
|  | CXCL11/CXCL10/CX3CL1 | GO:0042379 | chemokine receptor binding | 0.001893 | 3 |
|  | IFI44L/MX2/MX1/GBP4/GBP5 | GO:0005525 | GTP binding | 0.002402 | 5 |
|  | CXCL11/CXCL10 | GO:0045236 | CXCR chemokine receptor binding | 0.002402 | 2 |
|  | IFI44L/MX2/MX1/GBP4/GBP5 | GO:0032561 | guanyl ribonucleotide binding | 0.002402 | 5 |
|  | IFI44L/MX2/MX1/GBP4/GBP5 | GO:0019001 | guanyl nucleotide binding | 0.002402 | 5 |
|  | MX2/MX1/GBP4/GBP5 | GO:0003924 | GTPase activity | 0.002765 | 4 |
|  | CXCL11/CXCL10/RTP4/CX3CL1 | GO:0001664 | G-protein coupled receptor binding | 0.003677 | 4 |
|  | IFIH1/DDX58/DDX60L | GO:0004386 | helicase activity | 0.008319 | 3 |
|  | CXCL11/CXCL10/CX3CL1 | GO:0005125 | cytokine activity | 0.022305 | 3 |
|  | IFIH1/DDX58 | GO:0003727 | single-stranded RNA binding | 0.022548 | 2 |
|  | CXCL11/CXCL10/CX3CL1 | GO:0005126 | cytokine receptor binding | 0.032518 | 3 |
| 12h | CXCL10/CXCL11 | GO:0045236 | CXCR chemokine receptor binding | 0.008587 | 2 |
|  | MX2/IFI44L/MX1/GBP4/GBP5 | GO:0005525 | GTP binding | 0.008587 | 5 |
|  | MX2/IFI44L/MX1/GBP4/GBP5 | GO:0032561 | guanyl ribonucleotide binding | 0.008587 | 5 |
|  | MX2/IFI44L/MX1/GBP4/GBP5 | GO:0019001 | guanyl nucleotide binding | 0.008587 | 5 |
|  | MX2/MX1/GBP4/GBP5 | GO:0003924 | GTPase activity | 0.008587 | 4 |
|  | CXCL10/CXCL11 | GO:0008009 | chemokine activity | 0.036133 | 2 |
|  | CXCL10/CXCL11 | GO:0042379 | chemokine receptor binding | 0.044762 | 2 |
|  | OASL/OAS1 | GO:0003725 | double-stranded RNA binding | 0.044762 | 2 |
|  | CXCL10/CXCL11/SECTM1 | GO:0005125 | cytokine activity | 0.048798 | 3 |
| 18h | CXCL10/CXCL11/CCL5 | GO:0008009 | chemokine activity | 0.003365 | 3 |
|  | CXCL10/CXCL11/CCL5 | GO:0042379 | chemokine receptor binding | 0.003365 | 3 |
|  | OASL/OAS1/DDX58 | GO:0003725 | double-stranded RNA binding | 0.003365 | 3 |
|  | MX2/IFI44L/GBP4/MX1/GBP5 | GO:0005525 | GTP binding | 0.003767 | 5 |
|  | CXCL10/CXCL11 | GO:0045236 | CXCR chemokine receptor binding | 0.003767 | 2 |
|  | MX2/IFI44L/GBP4/MX1/GBP5 | GO:0032561 | guanyl ribonucleotide binding | 0.003767 | 5 |
|  | MX2/IFI44L/GBP4/MX1/GBP5 | GO:0019001 | guanyl nucleotide binding | 0.003767 | 5 |
|  | CXCL10/CXCL11/TNFSF13B/CCL5 | GO:0005125 | cytokine activity | 0.003767 | 4 |
|  | MX2/GBP4/MX1/GBP5 | GO:0003924 | GTPase activity | 0.003962 | 4 |
|  | CXCL10/CXCL11/TNFSF13B/CCL5 | GO:0005126 | cytokine receptor binding | 0.006333 | 4 |
|  | RSAD2/CCL5 | GO:0043621 | protein self-association | 0.016659 | 2 |

**Table S5** Top 30 Gene ontology (GO) analysis for H5N1 infected HTBE cells

|  | H5N1 | ID | Description | p.adjust | Count |
| --- | --- | --- | --- | --- | --- |
| 03h | KMT2D/KMT2A/SETD1B | GO:0042800 | histone methyltransferase activity (H3-K4 specific) | 0.00045 | 3 |
|  | KMT2D/KMT2A/SETD1B | GO:0018024 | histone-lysine N-methyltransferase activity | 0.002905 | 3 |
|  | KMT2D/KMT2A/SETD1B | GO:0016279 | protein-lysine N-methyltransferase activity | 0.002905 | 3 |
|  | KMT2D/KMT2A/SETD1B | GO:0016278 | lysine N-methyltransferase activity | 0.002905 | 3 |
|  | KMT2D/KMT2A/SETD1B | GO:0042054 | histone methyltransferase activity | 0.002905 | 3 |
|  | KDM6B/EP400/EGR1/EP300/ARID1A/KMT2A | GO:0003682 | chromatin binding | 0.002905 | 6 |
|  | KMT2D/KMT2A/SETD1B | GO:0008276 | protein methyltransferase activity | 0.006459 | 3 |
|  | KMT2D/KMT2A/SETD1B | GO:0008170 | N-methyltransferase activity | 0.006724 | 3 |
|  | KMT2D/KMT2A/SETD1B | GO:0008757 | S-adenosylmethionine-dependent methyltransferase activity | 0.025171 | 3 |
| 06h | CXCL11/CXCL10/CCL5 | GO:0008009 | chemokine activity | 0.003302 | 3 |
|  | CXCL11/CXCL10/CCL5 | GO:0042379 | chemokine receptor binding | 0.003302 | 3 |
|  | CXCL11/CXCL10/CCL5/IL16 | GO:0005125 | cytokine activity | 0.0053 | 4 |
|  | CXCL11/CXCL10 | GO:0045236 | CXCR chemokine receptor binding | 0.0053 | 2 |
|  | OASL/NCOA7/CCDC62 | GO:0035257 | nuclear hormone receptor binding | 0.012097 | 3 |
|  | OASL/NCOA7/CCDC62 | GO:0051427 | hormone receptor binding | 0.015703 | 3 |
|  | NCOA7/CCDC62 | GO:0030374 | ligand-dependent nuclear receptor transcription coactivator activity | 0.027578 | 2 |
| 12h | CXCL10/CXCL11/CCL5 | GO:0008009 | chemokine activity | 0.002705 | 3 |
|  | CXCL10/CXCL11/CCL5 | GO:0042379 | chemokine receptor binding | 0.002705 | 3 |
|  | CXCL10/CXCL11 | GO:0045236 | CXCR chemokine receptor binding | 0.006067 | 2 |
|  | NCOA7/CCDC62/OASL | GO:0035257 | nuclear hormone receptor binding | 0.012428 | 3 |
|  | NCOA7/CCDC62/OASL | GO:0051427 | hormone receptor binding | 0.015501 | 3 |
|  | RSAD2/CCL5 | GO:0043621 | protein self-association | 0.021584 | 2 |
|  | NCOA7/CCDC62 | GO:0030374 | ligand-dependent nuclear receptor transcription coactivator activity | 0.02371 | 2 |
|  | CXCL10/CXCL11/CCL5 | GO:0005125 | cytokine activity | 0.030085 | 3 |
|  | RASD1/RND1/GBP4 | GO:0003924 | GTPase activity | 0.03031 | 3 |
|  | CXCL10/CXCL11/CCL5 | GO:0001664 | G-protein coupled receptor binding | 0.036821 | 3 |
|  | CXCL10/CXCL11/CCL5 | GO:0005126 | cytokine receptor binding | 0.038026 | 3 |
| 18h | CXCL10/CXCL11/CCL5 | GO:0008009 | chemokine activity | 0.003809 | 3 |
|  | CXCL10/CXCL11/CCL5 | GO:0042379 | chemokine receptor binding | 0.003809 | 3 |
|  | CXCL10/CXCL11 | GO:0045236 | CXCR chemokine receptor binding | 0.007797 | 2 |
|  | CXCL10/CXCL11/CRISPLD2 | GO:0008201 | heparin binding | 0.032654 | 3 |
|  | RSAD2/CCL5 | GO:0043621 | protein self-association | 0.033211 | 2 |
|  | NCOA7/CCDC62 | GO:0030374 | ligand-dependent nuclear receptor transcription coactivator activity | 0.035451 | 2 |
|  | CXCL10/CXCL11/CRISPLD2 | GO:0005539 | glycosaminoglycan binding | 0.039124 | 3 |
|  | CXCL10/CXCL11/CCL5 | GO:0005125 | cytokine activity | 0.041008 | 3 |
|  | CXCL10/CXCL11/CRISPLD2 | GO:1901681 | sulfur compound binding | 0.041008 | 3 |
