# Supplementary material for: Differential responses of innate immunity triggered by different subtypes of influenza a viruses in human and avian hosts
Source: BMC Med Genomics. 2017 Dec 21;10(Suppl 4):70. doi: 10.1186/s12920-017-0304-z (PMC5763291; doi:10.1186/s12920-017-0304-z)
Supplement: Supplementary file 5 — Table S6. Top 10 up-regulated differentially expressed genes (DEGs) in ileum and lung of chicken infected with H5N1 and H5N2. Table S7. Top 10 up-regulated differentially expressed genes (DEGs) in ileum and lung of quail infected with H5N1 and H5N2 (DOCX 27 kb) [file 12920_2017_304_MOESM5_ESM.docx]

**Table S6.** Top 10 up-regulated differentially expressed genes (DEGs) in ileum and lung of chicken infected with H5N1 and H5N2

| **H5N1 chicken ileum 1d** | | | | | **H5N2 chicken ileum 1d** | | | | |
| --- | --- | --- | --- | --- | --- | --- | --- | --- | --- |
| gene | value_1 | value_2 | log2.fold_change. | p_value | gene | value_1 | value_2 | log2.fold_change. | p_value |
| CASQ2 | 0 | 26.2093 | Inf | 5.00E-05 | MMP7 | 0 | 6.70315 | Inf | 5.00E-05 |
| CACNG1 | 0 | 8.93386 | Inf | 5.00E-05 | APOV1 | 0 | 8.47711 | Inf | 5.00E-05 |
| MYL1 | 0 | 424.531 | Inf | 5.00E-05 | MIR6686 | 0 | 268.825 | Inf | 0.0011 |
| CKMT2 | 0 | 11.1808 | Inf | 5.00E-05 | APOH | 0 | 189.534 | Inf | 5.00E-05 |
| MYH1A,MYH1B,MYH1C,MYH1E | 1.16882 | 101.242 | 6.43661 | 5.00E-05 | ELOVL2 | 0 | 11.0124 | Inf | 5.00E-05 |
| TNNT3 | 3.05795 | 248.354 | 6.34369 | 5.00E-05 | CYP7A1 | 0 | 4.7803 | Inf | 5.00E-05 |
| IDS | 22.3737 | 992.265 | 5.47085 | 5.00E-05 | APOC3 | 0 | 2548.9 | Inf | 5.00E-05 |
| MYOM2 | 0.441451 | 11.4328 | 4.69478 | 5.00E-05 | LOC421740 | 0 | 5.79125 | Inf | 5.00E-05 |
| EEF1A2 | 2.63958 | 48.5007 | 4.19963 | 5.00E-05 | GAL8 | 0 | 14.2796 | Inf | 5.00E-05 |
| TNNC1 | 16.7529 | 156.691 | 3.22544 | 5.00E-05 | F9 | 0 | 10.9571 | Inf | 5.00E-05 |
| **H5N1 chicken ileum 3d** | | | | | **H5N2 chicken ileum 3d** | | | | |
| gene | value_1 | value_2 | log2.fold_change. | p_value | gene | value_1 | value_2 | log2.fold_change. | p_value |
| HMGA2 | 0 | 27.2647 | Inf | 5.00E-05 | AVD | 15.4268 | 341.004 | 4.46628 | 5.00E-05 |
| FOS | 136.882 | 1145.46 | 3.06492 | 5.00E-05 | PROKR2 | 1.09168 | 23.7983 | 4.44623 | 2.00E-04 |
| RASD1 | 4.47127 | 34.7069 | 2.95647 | 5.00E-05 | IL4I1 | 0.359411 | 5.40923 | 3.91172 | 0.0035 |
| ENS-1,ERNI | 9.99788 | 76.7212 | 2.93993 | 5.00E-05 | FGA | 2.10838 | 28.3111 | 3.74716 | 5.00E-05 |
| AREGB | 4.74182 | 33.8861 | 2.83718 | 5.00E-05 | KCNA3 | 1.36237 | 15.9865 | 3.55267 | 0.00175 |
| EGR1 | 125.39 | 757.296 | 2.59444 | 0.00035 | IL21R | 1.59739 | 17.9195 | 3.48774 | 5.00E-05 |
| SIK1 | 37.039 | 207.082 | 2.48308 | 5.00E-05 | CD72 | 1.67159 | 16.9547 | 3.34239 | 7.00E-04 |
| CYR61 | 121.335 | 628.06 | 2.37191 | 5.00E-05 | OLFM4 | 3.06772 | 30.7017 | 3.32308 | 5.00E-05 |
| LOC417536 | 97.2822 | 463.294 | 2.25168 | 1.00E-04 | SERPINA4 | 0.83598 | 7.89362 | 3.23915 | 0.00465 |
| SOCS3 | 8.80889 | 37.3616 | 2.08452 | 5.00E-05 | ALB | 55.088 | 493.579 | 3.16347 | 5.00E-05 |
| **H5N1 chicken lung 1d** | | | | | **H5N2 chicken lung 1d** | | | | |
| gene | value_1 | value_2 | log2.fold_change. | p_value | gene | value_1 | value_2 | log2.fold_change. | p_value |
| SCD | 12.3065 | 67.678 | 2.45927 | 5.00E-05 | ORM1 | 0 | 36.4469 | Inf | 5.00E-05 |
| FABP4 | 162.419 | 859.601 | 2.40395 | 5.00E-05 | AMBP | 0 | 6.532 | Inf | 5.00E-05 |
| APOA4 | 32.0345 | 161.132 | 2.33055 | 5.00E-05 | APOC3 | 0 | 133.609 | Inf | 1.00E-04 |
| THRSP | 15.3522 | 70.5624 | 2.20045 | 5.00E-05 | ALB | 4.2702 | 380.306 | 6.47671 | 5.00E-05 |
| CALB1 | 64.1686 | 276.092 | 2.10521 | 5.00E-05 | IDS | 18.7324 | 342.839 | 4.19393 | 5.00E-05 |
| PLIN1 | 17.4744 | 73.1734 | 2.06608 | 5.00E-05 | FGB | 1.25139 | 20.018 | 3.99969 | 5.00E-05 |
| SSTR2 | 2.11261 | 8.73563 | 2.04789 | 1.00E-04 | TTR | 7.36776 | 53.4904 | 2.85998 | 5.00E-05 |
| NPAS2 | 27.9886 | 113.47 | 2.0194 | 5.00E-05 | THRSP | 15.5281 | 79.6326 | 2.35848 | 5.00E-05 |
| LPL | 15.225 | 61.0747 | 2.00413 | 5.00E-05 | SCD | 12.4573 | 58.9467 | 2.24242 | 5.00E-05 |
| G0S2 | 19.2104 | 69.5727 | 1.85663 | 5.00E-05 | PSPH | 11.0038 | 49.4101 | 2.1668 | 0.00015 |
| **H5N1 chicken lung 3d** | | | | | **H5N2 chicken lung 3d** | | | | |
| gene | value_1 | value_2 | log2.fold_change. | p_value | gene | value_1 | value_2 | log2.fold_change. | p_value |
| IFNL3 | 0 | 9.09751 | Inf | 5.00E-05 | ALB | 1.88724 | 19.5701 | 3.3743 | 5.00E-05 |
| LYG2 | 2.35706 | 480.506 | 7.67142 | 5.00E-05 | CYP2C45 | 12.1574 | 42.0666 | 1.79084 | 5.00E-05 |
| TMEM173 | 14.5695 | 461.294 | 4.98466 | 1.00E-04 | IFI6 | 647.194 | 1778.25 | 1.45818 | 5.00E-05 |
| LOC417192 | 1.78618 | 47.4951 | 4.73283 | 1.00E-04 | MX1 | 51.3087 | 114.556 | 1.15878 | 5.00E-05 |
| SOCS1 | 11.551 | 300.897 | 4.70318 | 5.00E-05 | LY6E | 216.913 | 432.295 | 0.994899 | 5.00E-05 |
| LOC100858381 | 9.83299 | 209.242 | 4.4114 | 5.00E-05 | PPDPF | 903.031 | 465.51 | -0.95596 | 1.00E-04 |
| MX1 | 50.6961 | 951.271 | 4.22991 | 0.00015 | PDLIM7 | 170.002 | 80.3245 | -1.08164 | 5.00E-05 |
| IFI6 | 639.579 | 11970.8 | 4.22625 | 5.00E-05 | SLC8A3 | 6.16436 | 2.81805 | -1.12925 | 5.00E-05 |
| LY6E | 214.26 | 3919.02 | 4.19306 | 5.00E-05 | CALB1 | 78.547 | 25.057 | -1.64834 | 5.00E-05 |
| LOC770077 | 3.39118 | 55.8363 | 4.04134 | 0.00035 | FZD6 | 31.5943 | 8.77215 | -1.84866 | 5.00E-05 |

**Table S7.** Top 10 up-regulated differentially expressed genes (DEGs) in ileum and lung of quail infected with H5N1 and H5N2

| **H5N1 quail ileum 1d** | | | | | **H5N2 quail ileum 1d** | | | | |
| --- | --- | --- | --- | --- | --- | --- | --- | --- | --- |
| gene | value_1 | value_2 | log2.fold_change. | p_value | gene | value_1 | value_2 | log2.fold_change. | p_value |
| LOC107305802 | 0 | 7.72187 | Inf | 5.00E-05 | LOC107320070 | 0 | 2.20275 | Inf | 5.00E-05 |
| LOC107324940 | 3.97719 | 141.21 | 5.14995 | 1.00E-04 | LOC107307897 | 7.33585 | 63.7219 | 3.11875 | 5.00E-05 |
| RBP2 | 15.3575 | 146.509 | 3.25397 | 5.00E-05 | LOC107324143 | 3.10466 | 26.078 | 3.07033 | 5.00E-05 |
| ANO5 | 7.00631 | 62.6889 | 3.16148 | 5.00E-05 | VILL | 0.530107 | 4.45079 | 3.06971 | 5.00E-05 |
| LOC107311219 | 3.77463 | 25.2004 | 2.73904 | 5.00E-05 | LOC107319648 | 57.7163 | 398.527 | 2.78763 | 5.00E-05 |
| LCT | 1.64009 | 10.3621 | 2.65947 | 5.00E-05 | LOC107315867 | 62.8747 | 419.902 | 2.7395 | 5.00E-05 |
| GIP | 5.92431 | 37.3523 | 2.65648 | 0.00045 | SLC34A2 | 1.05246 | 6.81088 | 2.69407 | 5.00E-05 |
| LOC107306747 | 13.3263 | 83.1232 | 2.64097 | 5.00E-05 | ANO1 | 4.33811 | 27.5535 | 2.6671 | 5.00E-05 |
| HAAO | 2.98297 | 16.8004 | 2.49368 | 5.00E-05 | LOC107307093 | 0.773647 | 4.85122 | 2.6486 | 5.00E-05 |
| LOC107321478 | 7.4666 | 40.8943 | 2.45338 | 5.00E-05 | HEPHL1 | 0.614822 | 3.79362 | 2.62533 | 5.00E-05 |
| **H5N1 quail ileum 3d** | | | | | **H5N2 quail ileum 3d** | | | | |
| gene | value_1 | value_2 | log2.fold_change. | p_value | gene | value_1 | value_2 | log2.fold_change. | p_value |
| LOC107323787 | 0 | 11.4323 | Inf | 0.00145 | UNC80 | 0.256785 | 2.83569 | 3.46506 | 5.00E-05 |
| SLC28A3 | 0.199975 | 2.60347 | 3.70255 | 0.001 | C3 | 1.56564 | 12.6926 | 3.01916 | 5.00E-05 |
| UNC80 | 0.264484 | 2.79682 | 3.40254 | 5.00E-05 | LOC107315867 | 29.4785 | 198.262 | 2.74967 | 5.00E-05 |
| LOC107315131 | 1.11797 | 11.7031 | 3.38793 | 5.00E-05 | LOC107319648 | 28.5576 | 190.898 | 2.74085 | 5.00E-05 |
| SLC22A31 | 0.488845 | 4.0961 | 3.0668 | 0.001 | LOC107307354,LOC107307355 | 2.78156 | 16.4844 | 2.56714 | 4.00E-04 |
| NRXN3 | 1.98359 | 15.4358 | 2.9601 | 5.00E-05 | LOC107314675 | 34.8829 | 200.16 | 2.52056 | 5.00E-05 |
| LRRC56 | 1.30667 | 8.70969 | 2.73672 | 5.00E-05 | PDE1B | 0.726963 | 3.88818 | 2.41914 | 3.00E-04 |
| ACOT12 | 3.90511 | 25.1623 | 2.68783 | 5.00E-05 | LOC107312337 | 0.858619 | 4.5141 | 2.39435 | 0.00015 |
| LOC107325652 | 3.03088 | 16.2259 | 2.42049 | 5.00E-05 | CHRM4 | 0.793483 | 4.12977 | 2.37979 | 2.00E-04 |
| LOC107307210 | 22.9397 | 115.024 | 2.32602 | 0.0011 | LOC107309939 | 1336.39 | 6952.06 | 2.3791 | 1.00E-04 |
| **H5N1 quail lung 1d** | | | | | **H5N2 quail lung 1d** | | | | |
| gene | value_1 | value_2 | log2.fold_change. | p_value | gene | value_1 | value_2 | log2.fold_change. | p_value |
| COL9A1 | 0.674375 | 8.94677 | 3.72974 | 5.00E-05 | CPB2 | 0 | 2.4134 | Inf | 5.00E-05 |
| LOC107308805 | 1.44112 | 18.8987 | 3.71302 | 0.00035 | LOC107305801 | 0 | 5.96795 | Inf | 5.00E-05 |
| LECT2 | 1.40713 | 16.9172 | 3.58766 | 5.00E-05 | LOC107307924 | 0 | 4.98438 | Inf | 0.00205 |
| LOC107312329 | 0.977639 | 11.5643 | 3.56423 | 5.00E-05 | LOC107310114 | 1.22942 | 106.541 | 6.43728 | 1.00E-04 |
| LOC107318311 | 0.733347 | 4.71522 | 2.68476 | 7.00E-04 | LOC107325879 | 4.52677 | 364.797 | 6.33247 | 5.00E-05 |
| LOC107311370 | 0.542524 | 2.93782 | 2.43699 | 0.00045 | ORM2 | 5.02422 | 326.586 | 6.02242 | 5.00E-05 |
| TMEM179 | 0.53091 | 2.86965 | 2.43433 | 5.00E-05 | FGB | 3.15819 | 204.644 | 6.01788 | 5.00E-05 |
| FZD10 | 0.875451 | 3.78452 | 2.11201 | 5.00E-05 | APOB | 0.150415 | 9.49903 | 5.98076 | 5.00E-05 |
| LOC107324457 | 18.8234 | 66.998 | 1.83159 | 5.00E-05 | LOC107308805 | 1.4048 | 73.9964 | 5.71902 | 7.00E-04 |
| ST6GAL2 | 5.69981 | 20.0638 | 1.81561 | 5.00E-05 | FGA | 2.34189 | 116.815 | 5.6404 | 5.00E-05 |
| **H5N1 quail lung 3d** | | | | | **H5N2 quail lung 3d** | | | | |
| gene | value_1 | value_2 | log2.fold_change. | p_value | gene | value_1 | value_2 | log2.fold_change. | p_value |
| MYBPC3 | 0.231329 | 12.5098 | 5.75697 | 5.00E-05 | LOC107312474 | 2.03218 | 13.4072 | 2.72191 | 1.00E-04 |
| MYL2 | 2.43751 | 114.88 | 5.55858 | 5.00E-05 | EPSTI1 | 21.8431 | 69.9253 | 1.67864 | 5.00E-05 |
| LOC107314384 | 0.838536 | 27.3824 | 5.02923 | 0.00015 | P2RX7 | 20.2982 | 57.3911 | 1.49948 | 1.00E-04 |
| TBX20 | 0.086123 | 1.80676 | 4.39087 | 3.00E-04 | ANO1 | 6.11056 | 1.98439 | -1.62261 | 0.00035 |
| BMP10 | 0.400267 | 6.87108 | 4.1015 | 0.001 | HSPB8 | 31.2908 | 8.82658 | -1.82581 | 1.00E-04 |
| FZD10 | 0.582833 | 4.45655 | 2.93477 | 0.00105 | GJA1 | 33.5535 | 9.22991 | -1.86208 | 3.00E-04 |
| LOC107312474 | 2.00938 | 14.4789 | 2.84913 | 5.00E-05 | PDK4 | 12.1806 | 3.3086 | -1.8803 | 0.00025 |
| RPH3A | 0.284247 | 1.97595 | 2.79733 | 0.00165 | NFKBIE | 27.7466 | 7.40837 | -1.90508 | 3.00E-04 |
| LOC107322603 | 1.05676 | 7.22569 | 2.77349 | 5.00E-05 | LOC107319878 | 11.4516 | 3.02588 | -1.92013 | 5.00E-05 |
| DYNC1I1 | 0.31091 | 1.93064 | 2.63451 | 1.00E-04 | ADAMTS3 | 11.8594 | 2.95878 | -2.00296 | 3.00E-04 |
