# Supplementary material for: Differential responses of innate immunity triggered by different subtypes of influenza a viruses in human and avian hosts
Source: BMC Med Genomics. 2017 Dec 21;10(Suppl 4):70. doi: 10.1186/s12920-017-0304-z (PMC5763291; doi:10.1186/s12920-017-0304-z)
Supplement: Supplementary file 6 — Table S8. Differentially expressed immune-related genes in chicken. Table S9. Differentially expressed immune-related genes in quail (DOCX 21 kb) [file 12920_2017_304_MOESM6_ESM.docx]

**Table S8** Differentially expressed immune-related genes in chicken

|  | H5N1 chicken ileum 1 dpi | | | | | | | | | | H5N1 chicken ileum 3 dpi | | | | | | | |
| --- | --- | --- | --- | --- | --- | --- | --- | --- | --- | --- | --- | --- | --- | --- | --- | --- | --- | --- |
| gene | log2(fold_change) | | | p_value | | | significant | | | | log2(fold_change) | | p_value | | significant | | | |
| IFI6 | -0.69507 | | | 0.0002 | | | yes | | | | - | | - | | - | | | |
| IFIH1 | -0.63441 | | | 0.0009 | | | yes | | | | - | | - | | - | | | |
| SOCS3 | - | | | - | | | - | | | | 2.08452 | | 5.00E-05 | | yes | | | |
|  | | | | | | | | | | | | | | | | | | |
|  | | H5N1 chicken lung 1 dpi | | | | | | | | | H5N1 chicken lung 3 dpi | | | | | | | |
| gene | | log2(fold_change) | | | p_value | | | significant | | | log2(fold_change) | | | p_value | | | significant | |
| IFNAR1 | | - | | | - | | | - | | | 2.32992 | | | 5.00E-05 | | | yes | |
| IFNL3 | | - | | | - | | | - | | | inf | | | 5.00E-05 | | | yes | |
| SOCS1 | | - | | | - | | | - | | | 4.70318 | | | 5.00E-05 | | | yes | |
| SOCS3 | | - | | | - | | | - | | | 2.10794 | | | 5.00E-05 | | | yes | |
| TLR3 | | - | | | - | | | - | | | 2.15801 | | | 0.0002 | | | yes | |
| MX1 | | - | | | - | | | - | | | 4.22991 | | | 0.00015 | | | yes | |
| IL16 | | - | | | - | | | - | | | 1.44474 | | | 0.00025 | | | yes | |
| IFI6 | | -1.41727 | | | 5.00E-05 | | | yes | | | - | | | - | | | - | |
| IL7R | | 0.842218 | | | 5.00E-05 | | | yes | | | - | | | - | | | - | |
| NFIL3 | | 0.826137 | | | 0.00015 | | | yes | | | - | | | - | | | - | |
|  | | | | | | | | | | | | | | | | | | |
|  | | | H5N2 chicken ileum 1 dpi | | | | | | | | | H5N1 chicken ileum 3 dpi | | | | | | |
| gene | | | log2(fold_change) | | | p_value | | | | significant | | log2(fold_change) | | p_value | | | significant | |
| IFNGR1 | | | - | | | - | | | | - | | -0.93869 | | 0.0001 | | | yes | |
| SOCS1 | | | 1.6344 | | | 0.0004 | | | | yes | | 1.5491 | | 0.0005 | | | yes | |
| IFIH1 | | | - | | | - | | | | - | | 1.1721 | | 5.00E-05 | | | yes | |
| MX1 | | | 1.93013 | | | 5.00E-05 | | | | yes | | 2.19157 | | 5.00E-05 | | | yes | |
| NFIL3 | | | - | | | - | | | | - | | 0.995133 | | 5.00E-05 | | | yes | |
| SOCS3 | | | 1.60142 | | | 0.0001 | | | | yes | | - | | - | | | - | |
| TLR7 | | | 1.82185 | | | 5.00E-05 | | | | yes | | - | | - | | | - | |
| TLR2-1 | | | 1.00277 | | | 0.00215 | | | | yes | | - | | - | | | - | |
| TLR1LB | | | 1.30168 | | | 0.0009 | | | | yes | | - | | - | | | - | |
| TLR1LA | | | 2.78694 | | | 5.00E-05 | | | | yes | | - | | - | | | - | |
| IFI6 | | | 0.649737 | | | 0.00245 | | | | yes | | - | | - | | | - | |
| IL18R1 | | | 1.86645 | | | 5.00E-05 | | | | yes | | - | | - | | | - | |
| IL2RA | | | 1.90035 | | | 0.0005 | | | | yes | | - | | - | | | - | |
| IL16 | | | 2.36166 | | | 5.00E-05 | | | | yes | | - | | - | | | - | |
| IL21R | | | 3.48774 | | | 5.00E-05 | | | | yes | | - | | - | | | - | |
| IL4I1 | | | 3.91172 | | | 0.0035 | | | | yes | | - | | - | | | - | |
| IL10RA | | | 1.96132 | | | 0.00015 | | | | yes | | - | | - | | | - | |
| IL18 | | | 1.62209 | | | 0.00075 | | | | yes | | - | | - | | | - | |
| IL2RG | | | 1.82369 | | | 5.00E-05 | | | | yes | | - | | - | | | - | |
| IL13RA2 | | | 1.64577 | | | 0.00445 | | | | yes | | - | | - | | | - | |
| IL7R | | | 1.75632 | | | 5.00E-05 | | | | yes | | - | | - | | | - | |
|  | | | | | | | | | | | | | | | | | | |
|  | | | H5N2 chicken lung 1 dpi | | | | | | | | | H5N1 chicken lung 3 dpi | | | | | |  |
| gene | | | log2(fold_change) | | | p_value | | | significant | | | log2(fold_change) | | p_value | | significant | |  |
| IFI6 | | | - | | | - | | | - | | | 1.45818 | | 5.00E-05 | | yes | |  |
| MX1 | | | - | | | - | | | - | | | 1.15878 | | 5.00E-05 | | yes | |  |

**Table S9** Differentially expressed immune-related genes in quail

|  | H5N1 quail ileum 1 dpi | | | H5N1 quail ileum 3 dpi | | |
| --- | --- | --- | --- | --- | --- | --- |
| gene | log2(fold_change) | p_value | significant | log2(fold_change) | p_value | significant |
| SOCS3 | -2.34183 | 5.00E-05 | yes |  |  |  |
| DHX58 | -1.38207 | 0.00115 | yes |  |  |  |
| IL34 | -2.27201 | 5.00E-05 | yes | -1.58446 | 5.00E-05 | yes |
| IL4I1 | - | - | - | -2.89291 | 0.0001 | yes |
|  | | | | | | |
|  | H5N1 quail lung 1 dpi | | | H5N1 quail lung 3 dpi | | |
| gene | log2(fold_change) | p_value | significant | log2(fold_change) | p_value | significant |
| SOCS3 | -1.68866 | 0.0001 | yes | -3.17568 | 5.00E-05 | yes |
| TLR3 | 0.986485 | 0.0001 | yes | - | - | - |
| IFIH1 | 0.812249 | 0.00535 | yes | - | - | - |
| IL1RL1 | 0.90752 | 0.0004 | yes | - | - | - |
| IL18R1 | 0.97176 | 0.0012 | yes | - | - | - |
| IL17REL | 1.13151 | 5.00E-05 | yes | - | - | - |
| IL18 | 1.32338 | 0.00025 | yes | - | - | - |
| SOCS3 | - | - | - | -3.17568 | 5.00E-05 | yes |
| TLR7 | - | - | - | -3.46848 | 5.00E-05 | yes |
| TLR4 | - | - | - | -3.29345 | 0.0001 | yes |
| IL2RB | - | - | - | -2.42861 | 0.0002 | yes |
| IL1R2 | - | - | - | -1.98279 | 0.0009 | yes |
| IL18R1 | - | - | - | -1.85394 | 0.00085 | yes |
| IL17RA | - | - | - | -2.28619 | 5.00E-05 | yes |
| IL22RA2 | - | - | - | -2.15395 | 0.00025 | yes |
| IL2RG | - | - | - | -3.24308 | 5.00E-05 | yes |
| IL16 | - | - | - | -2.97749 | 5.00E-05 | yes |
| IL21R | - | - | - | -3.7028 | 0.00025 | yes |
| IL10RA | - | - | - | -2.39543 | 0.00045 | yes |
| IL7R | - | - | - | -3.06294 | 5.00E-05 | yes |
|  | | | | | | |
|  | H5N2 quail ileum 1 dpi | | | H5N2 quail ileum 3 dpi | | |
| gene | log2(fold_change) | p_value | gene | log2(fold_change) | p_value | gene |
| SOCS1 | 1.44317 | 0.00015 | SOCS1 | 1.44317 | 0.00015 | SOCS1 |
| DHX58 | 1.51685 | 5.00E-05 | DHX58 | 1.51685 | 5.00E-05 | DHX58 |
| IFIH1 | 1.15531 | 5.00E-05 | IFIH1 | 1.15531 | 5.00E-05 | IFIH1 |
| IL20RA | 1.45495 | 5.00E-05 | IL20RA | 1.45495 | 5.00E-05 | IL20RA |
| IL34 | -1.67816 | 5.00E-05 | IL34 | -1.67816 | 5.00E-05 | IL34 |
|  | | | | | | |
|  | H5N2 quail lung 1 dpi | | | H5N2 quail lung 3 dpi | | |
| gene | log2(fold_change) | p_value | significant | log2(fold_change) | p_value | significant |
| SOCS1 | 1.64117 | 0.0002 | yes | - | - | - |
| SOCS3 | 1.14411 | 0.0009 | yes | - | - | - |
| TLR7 | 1.75482 | 0.0003 | yes | - | - | - |
| TLR3 | 1.5425 | 5.00E-05 | yes | - | - | - |
| TLR4 | 2.21035 | 0.0002 | yes | - | - | - |
| IFIH1 | 2.08137 | 5.00E-05 | yes | - | - | - |
| DHX58 | 2.20096 | 5.00E-05 | yes | - | - | - |
| CXCL12 | -2.26972 | 5.00E-05 | yes | - | - | - |
| ILDR2 | -1.52728 | 5.00E-05 | yes | - | - | - |
| IL1R2 | 2.52966 | 5.00E-05 | yes | - | - | - |
| IL17REL | -1.81236 | 5.00E-05 | yes | - | - | - |
| IL22RA2 | 2.48139 | 5.00E-05 | yes | - | - | - |
| IL20RA | 0.916439 | 0.0027 | yes | - | - | - |
| IL13RA1 | 0.869103 | 0.0025 | yes | - | - | - |
| IL15 | 1.50985 | 0.0021 | yes | - | - | - |
| IL16 | 1.71523 | 0.0005 | yes | - | - | - |
| IL11RA | -1.43995 | 0.0001 | yes | - | - | - |
| NFIL3 | 1.16181 | 5.00E-05 | yes | - | - | - |
