# Supplementary material for: Differential responses of innate immunity triggered by different subtypes of influenza a viruses in human and avian hosts
Source: BMC Med Genomics. 2017 Dec 21;10(Suppl 4):70. doi: 10.1186/s12920-017-0304-z (PMC5763291; doi:10.1186/s12920-017-0304-z)

**Figure S2.** Expression profiles of IFNs in HBE cells infected with H1N1.


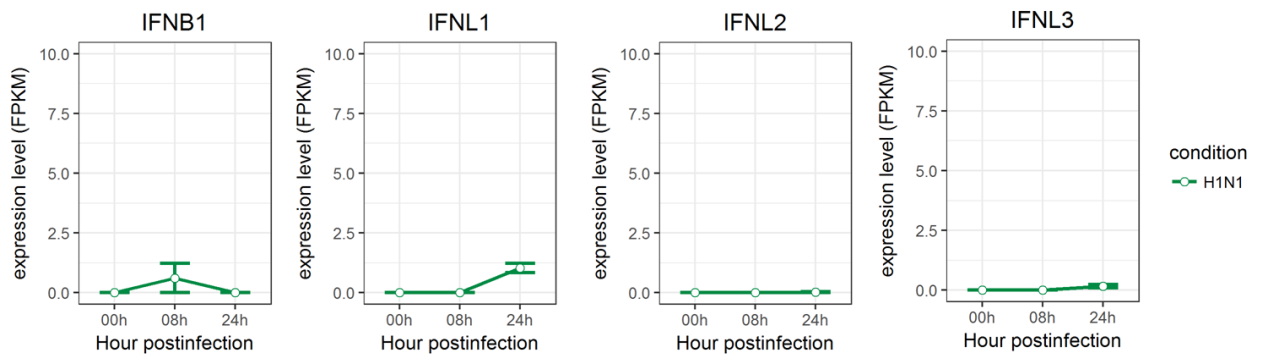


**Figure S3.** Expression profiles of IFN-stimulated genes in HBE cells infected with H1N1.


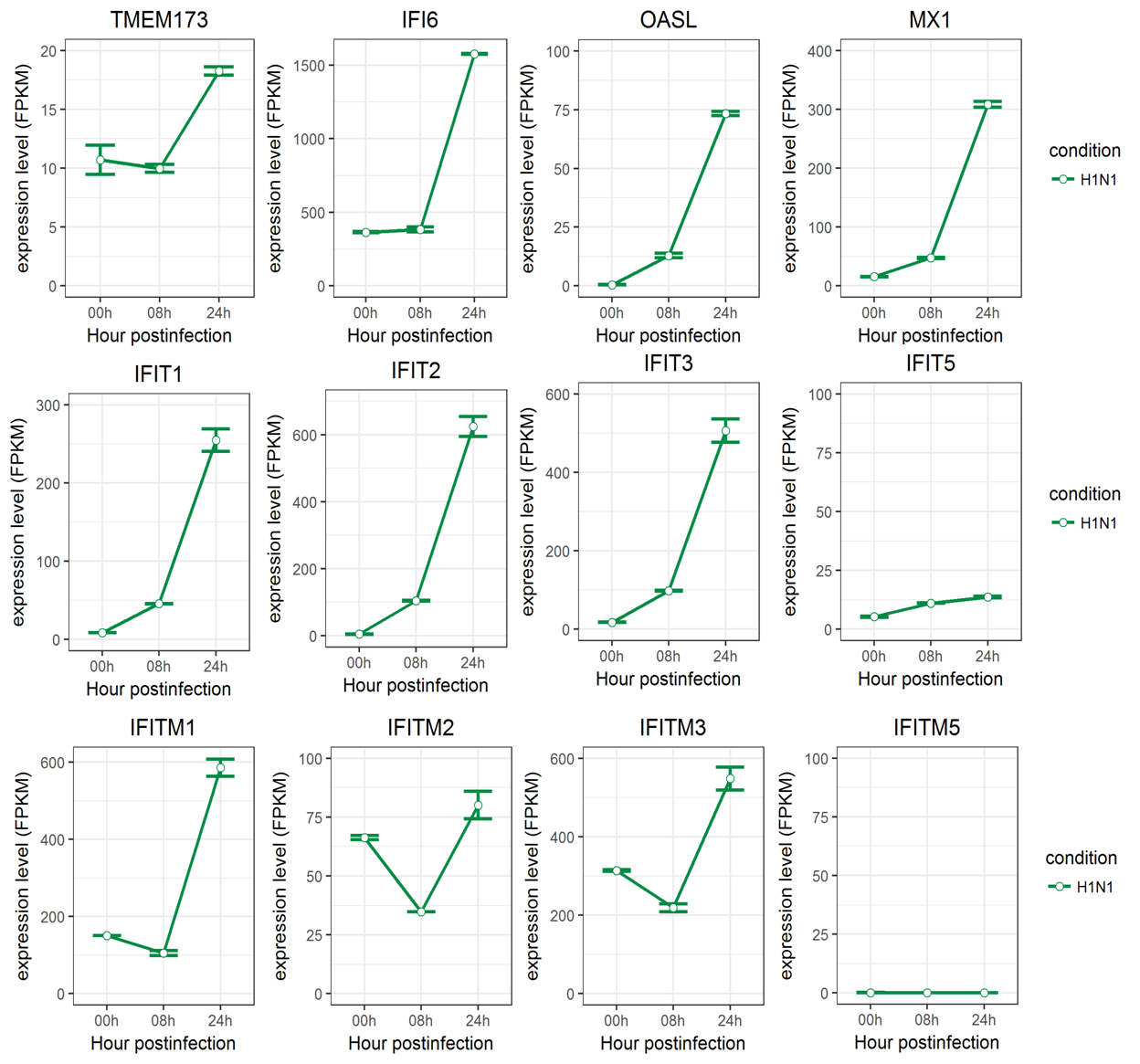


**Figure S4.** Expression profiles of C-X-C motif ligands, C-C motif ligands in HBE cells infected with H1N1.


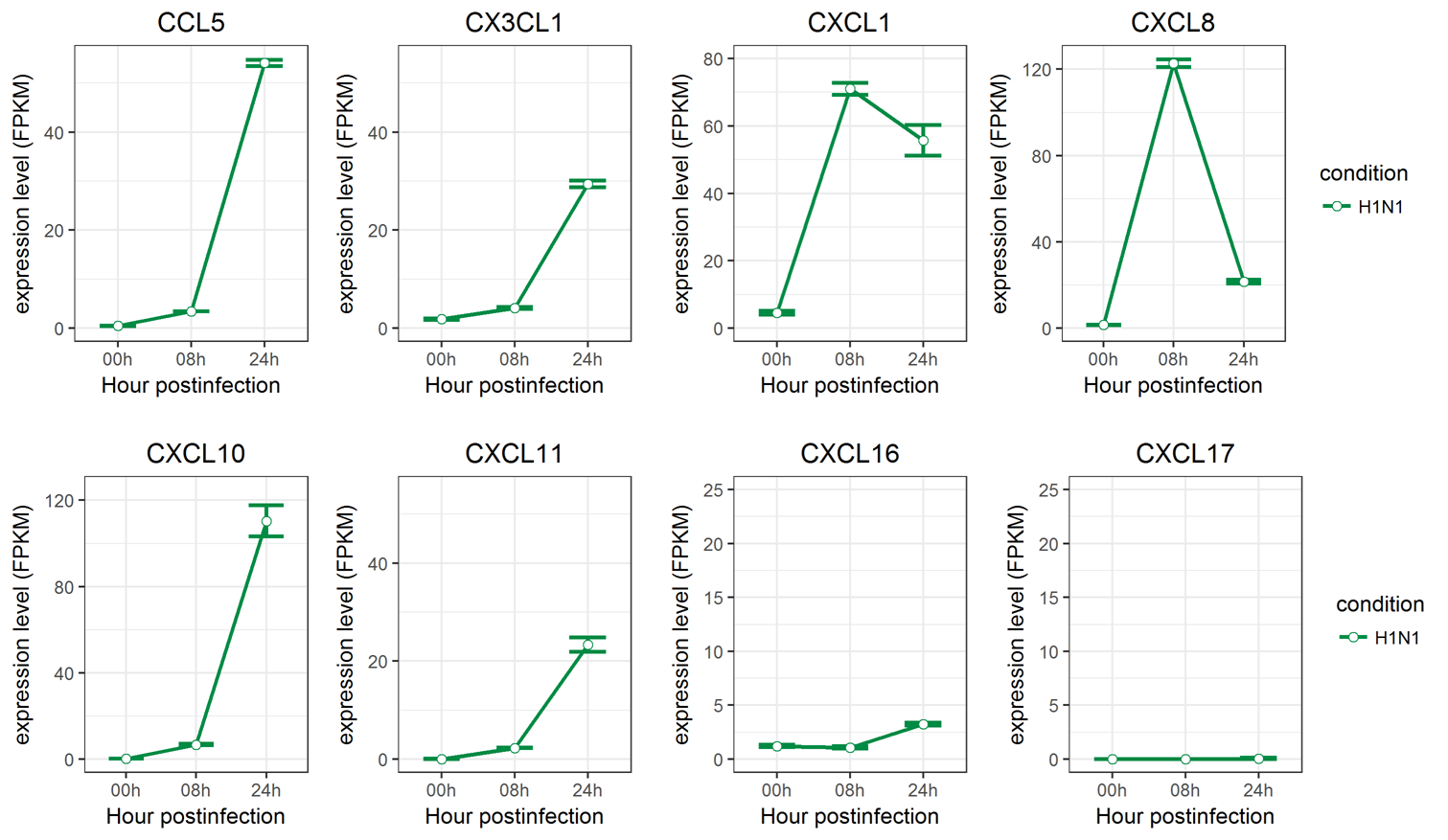


**Figure S4.** Expression profiles of suppressors of cytokines in HBE cells infected with H1N1.


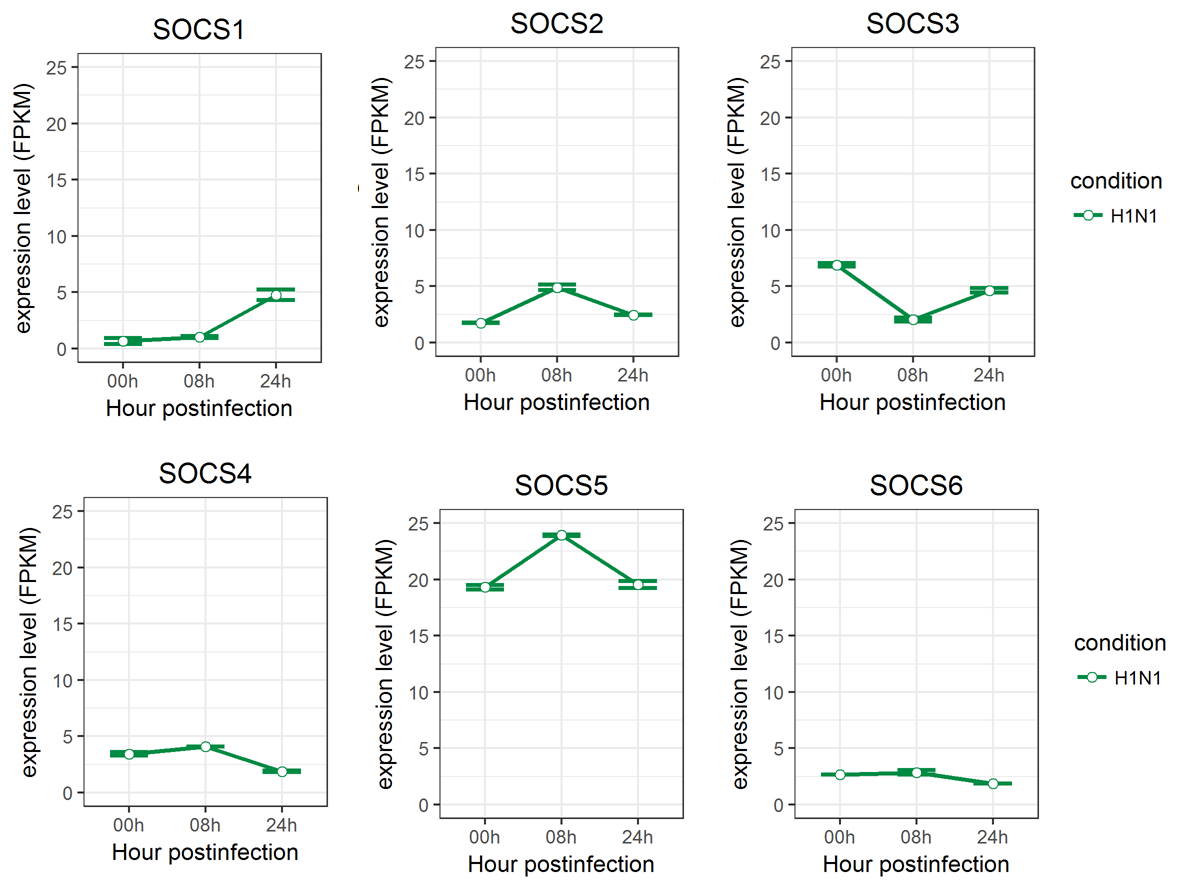

Supplement: Supplementary file 7 — Fig. S1. Work flow of this study (DOCX 837 kb) [file 12920_2017_304_MOESM7_ESM.docx]
